# Protoflavone-Chalcone Hybrids Exhibit Enhanced Antitumor Action through Modulating Redox Balance, Depolarizing the Mitochondrial Membrane, and Inhibiting ATR-Dependent Signaling

**DOI:** 10.3390/antiox9060519

**Published:** 2020-06-12

**Authors:** Ahmed Dhahir Latif, Tamás Jernei, Ana Podolski-Renić, Ching-Ying Kuo, Máté Vágvölgyi, Gábor Girst, István Zupkó, Sedef Develi, Engin Ulukaya, Hui-Chun Wang, Milica Pešić, Antal Csámpai, Attila Hunyadi

**Affiliations:** 1 Institute of Pharmacodynamics and Biopharmacy, Interdisciplinary Excellence Centre, University of Szeged, Eötvös str. 6, H-6720 Szeged, Hungary; latif.ahmed@pharmacognosy.hu (A.D.L.); zupko@pharm.u-szeged.hu (I.Z.); 2 Institute of Pharmacognosy, Interdisciplinary Excellence Centre, University of Szeged, Eötvös str. 6, H-6720 Szeged, Hungary; vagvolgyi.mate@pharmacognosy.hu (M.V.); girst.gabor@pharmacognosy.hu (G.G.); 3 Institute of Chemistry, Eötvös Loránd University, P.O. Box 32, H-1518 Budapest-112, Hungary; jernei@caesar.elte.hu; 4 Department of Neurobiology, Institute for Biological Research “Siniša Stanković”- National Institute of Republic of Serbia, University of Belgrade, Bulevar Despota Stefana 142, 11060 Belgrade, Serbia; ana.podolski@ibiss.bg.ac.rs (A.P.-R.); camala@ibiss.bg.ac.rs (M.P.); 5 Graduate Institute of Natural Products, Kaohsiung Medical University, Shih-Chuan 1st Rd. 100, Kaohsiung 807, Taiwan; r980279@kmu.edu.tw (C.-Y.K.); wanghc@kmu.edu.tw (H.-C.W.); 6 Interdisciplinary Centre for Natural Products, University of Szeged, Eötvös str. 6, H-6720 Szeged, Hungary; 7 Molecular Cancer Research Center, Istinye University, 34010 Topkapi, Istanbul, Turkey; elif.develi@stu.istinye.edu.tr (S.D.); eulukaya@istinye.edu.tr (E.U.)

**Keywords:** antitumor natural product, protoflavone, chalcone, ferrocene, hybrid compound, fragment-based drug design, DNA damage response, oxidative stress, virtual combination study

## Abstract

Hybrid compounds combine fragments with complementary targets to achieve a common pharmacological goal. This approach represents an increasingly popular strategy for drug discovery. In this work, we aimed to design antitumor hybrid compounds based on an inhibitor of ataxia-telangiectasia and Rad3-related protein (ATR)-dependent signaling, protoapigenone, and a pro-oxidant ferrocene or chalcone fragment. Four new triazole-coupled hybrids were prepared. The compounds were cytotoxic against human breast cancer cell lines in vitro, showing IC_50_ values in the sub-micromolar range. The nature of interactions between relevant fragments of the hybrids was evaluated by the Chou–Talalay method. Experimental combination treatment with the fragments showed additive effects or slight/moderate synergism, while strong synergism was observed when the fragments were virtually combined into their hybrids, suggesting a relevant pharmacological benefit of the coupling. All hybrids were strong inhibitors of the ATR-mediated activation of Chk1, and they interfered with the redox balance of the cells leading to mitochondrial membrane depolarization. Additionally, they induced late apoptosis and primary necrosis in MDA-MB-231 and MCF-7 breast cancer cells, respectively. Our results demonstrate that coupling the ATR-dependent signaling inhibitor protoflavone with a pro-oxidant chalcone dramatically increases the antitumor activity compared with either fragment alone. Such compounds may offer an attractive novel strategy for the treatment of various cancers.

## 1. Introduction

Affecting 2.1 million people each year, breast cancer is the most common type of cancer among women, and it is one of the leading causes of cancer-related deaths. It is estimated that more than 600,000 women died of breast cancer in 2018, accounting for about 15% of all cancer deaths among women [1]. If diagnosed early, it is highly curable. However, the five-year survival rate of patients with metastatic breast cancer is poor. More devastatingly, almost a fifth of patients will develop local or distant recurrence within five years of diagnosis [2]. Therefore, new treatment options are essential, of which novel anticancer compounds are still desperately needed. This is particularly true for triple-negative breast cancer (TNBC), the most challenging breast cancer subtype to treat. Since it is resistant to first-line antiestrogen therapy, its current treatment options are limited and require the use of chemotherapeutic agents [3,4].

For fragment-based drug discovery, the design and preparation of hybrid compounds is an attractive strategy that has been increasingly popular in the search for new multitarget drugs, particularly against multifactorial chronic diseases [5,6,7]. In this context, molecular hybridization is also regarded as a highly efficient strategy to produce multitarget drug candidates with significantly enhanced anticancer activity, often due to a synergism associated with the cooperative effects of the individual molecular fragments.

Typically found in fern species (e.g., *Thelypteris* and *Pseudophegopteris*), protoflavones are rare natural flavonoids with an unusual non-aromatic B-ring [8]. Several of these compounds have been found to exert promising antitumor effects on a wide variety of cell lines in vitro [9,10,11,12,13] and various xenograft models in vivo [12,14,15,16]. Protoflavonoids were reported to induce apoptosis and cause S and G2/M phase cell cycle arrest [17,18], and this is closely associated with their ability to induce oxidative stress [19]. It is of particular interest that protoapigenone, the protoflavone analog of apigenin, is a potent inhibitor of the ataxia-telangiectasia and Rad3-related protein (ATR)-mediated activation of checkpoint kinase 1 (Chk-1), a crucial component of the replication-associated DNA damage response (DDR) [16]. Along with ataxia-telangiectasia mutated (ATM) kinase, this pathway is an attractive novel antitumor target that is currently being investigated in several related clinical trials [20,21,22]. Playing an essential role in the regulation of DNA repair pathways, ATR and ATM are activated upon DNA damage induced by oxidative stress [23]. Therefore, simultaneously targeting DDR and inducing oxidative stress may be a relevant antitumor strategy.

While protoflavones themselves already join these two pharmacological properties, we aimed to further exploit this by preparing hybrid compounds of a protoflavone (i.e., ATR inhibitor and oxidative stress inducer) and chalcones representing another type of fragment that may induce oxidative stress and are capable of uncoupling mitochondrial respiration to inhibit mitochondrial membrane potential [24]. Through a variety of molecular mechanisms, as discussed in recent reviews, chalcones have significant potential to cause cytotoxic effects against cancer cell lines [25,26,27]. In this context, we previously prepared antiproliferative hybrids comprising ferrocenyl-substituted chalcone and cinchona residues connected with triazole linkers, which were found to display marked cytotoxic activity against human liver cancer (HepG2) and human colon cancer (HT-29) cell lines [28]. Significantly, two representatives of these hybrids were found to act as pro-oxidants sensitizing three multidrug-resistant (MDR) human cancer cell lines and their sensitive counterparts (non-small cell lung carcinoma NCI-H460/R/NCI-H460, colorectal carcinoma DLD1-TxR/DLD1, and glioblastoma U87-TxR/U87) to paclitaxel [29]. We also established that these ferrocene-containing hybrids are not substrates for *P*-glycoprotein, a drug transporter responsible for the MDR phenotype, and that they increase the production of reactive oxygen species (ROS), leading to mitochondrial damage in MDR cancer cells.

As a continuation of our search for potent anticancer hybrids with enhanced activity, we performed the synthesis, in vitro evaluation, and mechanistic study of novel hybrids containing a protoflavone and a chalcone residue. Both moieties may serve as promising building blocks of antitumor agents influencing redox homeostasis [30,31,32]. Therefore, in this study, we aimed to investigate if the addition of a potentially pro-oxidant fragment, such as a chalcone, can further enhance the antitumor activity of a protoflavone while maintaining or possibly further improving its inhibitory effect on ATR-mediated signaling.

## 2. Materials and Methods

### 2.1. General

All fine chemicals for synthesis were obtained from commercially available sources (Merck, Fluorochem, Molar Chemicals, VWR) and were used without further purification. Merck Kieselgel (230–400 mesh, 60 Ǻ) was used for flash column chromatography. The ^1^H- and ^13^C-NMR spectra of all of the compounds were recorded in CDCl_3_ or dimethyl sulfoxide (DMSO)-*d*_6_ solution in 5 mm tubes at room temperature on a Bruker DRX-500 spectrometer at 500 (^1^H) and 125 (^13^C) MHz with the deuterium signal of the solvent as the lock and TMS as the internal standard. The HSQC and HMBC spectra, which support the exact assignment of ^1^H- and ^13^C NMR signals, were obtained using the standard Bruker pulse programs. HRMS spectra were recorded on a Q Exactive Plus hybrid quadrupole-orbitrap mass spectrometer (Thermo Scientific, Waltham, MA, USA) equipped with a heated electrospray ionization (HESI-II) probe that was used in the positive mode, and flow injection analysis was performed by an Abi 140C Syringe Pump.

For bioactivity assays, 96% ethanol was obtained from Molar Chemicals Ltd. (Halásztelek, Hungary). Dimethyl sulfoxide (DMSO) was purchased from Fisher Scientific Co. UK, Trypan Blue Solution was from Mediatech. Co. USA, Trypsin Replacement Enzyme was from Life Technologies UK, and cisplatin was obtained from Accord Healthcare France SAS. Penicillin-streptomycin-amphotericin B, non-essential amino acid (NEAA) mixture, phosphate-buffered saline (PBS), and minimal essential medium (MEM) were purchased from Lonza, Walkersville, USA. All plates, flasks, and cell culture vessels were purchased from Biologix Europe GmbH. Propidium iodide (PI), 3-(4, 5-dimethylthiazol-2-yl)-2, 5-diphenyltetrazolium bromide (MTT), Triton-X-100, ribonuclease A (RNase A), fetal bovine serum (FBS), the caspase 3 colorimetric assay kit and all other reagents, solvents, and chemicals were obtained from Sigma-Aldrich Co. (St. Louis, MO, USA).

### 2.2. Synthesis of Compound ***1*** from Apigenin

Protoapigenone 1′-*O*-propargyl ether (**1**) was synthesized as previously published [33]. Briefly, apigenin was dissolved to obtain a 1 mg/mL concentration in a 9:1 mixture of acetonitrile and propargyl alcohol. While stirring, two equivalents of [bis(trifluoroacetoxy)iodo]benzene were slowly added to the solution, and the reaction was left to develop for 1 h at 80 °C. After completion of the reaction, the mixture was cooled down, the solvent was evaporated under reduced pressure on a rotary evaporator, and the crude mixture was re-dissolved in methanol and adsorbed on silica. For purification, flash chromatography was performed on a CombiFlash Rf+ instrument (Teledyne Isco, Lincoln, NE, USA) with a 40 g HP Silica RediSep Rf Gold column (Teledyne Isco, Lincoln, NE, USA) with the eluent of the mixture of *n*-hexane:ethyl acetate:acetone (12:3:1, *v*/*v*/*v*). The reaction was repeated several times from a total of 1 g of apigenin, and the overall final isolated yield was 30%.

### 2.3. Synthesis of Hybrid Compounds ***3a**–**d***

The corresponding azide (**2a**–**d**; 0.31 mmol, 1.0 equation), 100 mg of 5,7-dihydroxy-2-(4-oxo-1-(prop-2-yn-1-yloxy)cyclohexa-2,5-dien-1-yl)-4*H*-chromen-4-one (**1**; 0.31 mmol, 1.0 equation), CuSO_4_ (14 mg, 0.06 mmol, 0.2 equation), and sodium ascorbate (61 mg, 0.31 mmol, 1.0 equation) were suspended in a 1:1 mixture of water and *n*-BuOH (1 mL). The reaction mixture was stirred at room temperature for 12 h, and then it was poured on brine (20 mL) and extracted with dichloromethane (DCM; 5 × 20 mL). The combined organic phases were dried over anhydrous Na_2_SO_4_, and the solvent was evaporated. The residue was purified by column chromatography on silica gel using a 20:1 mixture of DCM and methanol as eluent to obtain the pure product. After chromatography, the analytical sample was crystallized from DCM. Yields and characterization, along with detailed assignments and copies of ^1^H- and ^13^C NMR spectra, are found in the Supporting Information (Figures S1–S8).

### 2.4. Synthesis of the Reference Fragment ***8a***

This ferrocene derivative was prepared in two consecutive steps, (**a**) and (**b**), as follows:

#### 2.4.1. (a) Synthesis of 2-(4-(Hydroxymethyl)-1*H*-1,2,3-triazol-1-yl)benzaldehyde *(**6**)*

Propargyl alcohol (**4**; 0.17 mL, 0.17 g, 3.0 mmol, 1.0 eq.), 2-azidobenzaldehyde (**5**; 0.44 g, 3.0 mmol, 1.0 eq.), CuSO_4_ (0.10 g, 0.6 mmol, 0.2 eq.), and sodium ascorbate (0.59 g, 3.0 mmol, 1.0 equation) were suspended in a 1:1 mixture of water and *n*-BuOH (1 mL). The reaction mixture was stirred at room temperature for 12 h, and then it was poured on water (20 mL) and extracted with DCM (5 × 20 mL). The combined organic phase was dried on anhydrous Na_2_SO_4_, and the solvent was evaporated. The crude product was purified by column chromatography on silica gel using DCM as the eluent to obtain **6** as a pure product of which the yield and characterization, along with detailed assignments and copies of ^1^H and ^13^C NMR spectra, are found in the Supporting Information (Figures S9 and S10).

#### 2.4.2. (b) Synthesis of (*E*)-1-Ferrocenyl-3-(2-(4-(hydroxymethyl)-1*H*-1,2,3-triazole-1-yl)phenyl)prop-2-en-1-one *(**8a**)*

2-(4-(Hydroxymethyl)-1*H*-1,2,3-triazole-1-yl)benzaldehyde (**6**; 0.20 g, 1.0 mmol, 1.0 eq.) and acetylferrocene (**7**; 0.23 g, 1.0 mmol, 1.0 eq.) were dissolved in 2 mL of EtOH. To this solution, 10% aqueous NaOH (0.2 mL) was added at room temperature, and the resulting mixture was stirred for 12 h and then poured on water (50 mL). The formed precipitate was collected by filtration, washed with water, dried, and subjected to column chromatography on a silica gel using DCM as the eluent to obtain **8a** as a pure product of which the yield and characterization, along with detailed assignments and copies of ^1^H and ^13^C NMR spectra, are found in the Supporting Information (Figures S11 and S12).

### 2.5. Synthesis of Reference Fragments ***8b**–**d***

The corresponding azide (**2b**–**d**; 1.0 mmol, 1.0 eq.), propargyl alcohol (**4**; 58 µL, 56 mg, 1.0 mmol, 1.0 eq.), CuSO_4_ (32 mg, 0.2 mmol, 0.2 eq.), and sodium ascorbate (0.20 g, 1.0 mmol, 1.0 eq.) were suspended in a 1:1 mixture of water and *n*-BuOH (4 mL). The reaction mixture was stirred at room temperature for 12 h, and then it was poured on water (20 mL) and extracted with DCM (5 × 20 mL). The combined organic phase was dried on anhydrous Na_2_SO_4_, and the solvent was evaporated. The crude product was sequentially purified by column chromatography on a silica gel using DCM:MeOH (20:1 and 10:1) mixtures as eluents. After purification, the analytical sample was crystallized from MeOH. Yields and characterization, along with detailed assignments and copies of ^1^H and ^13^C NMR spectra, are provided as Supporting Information (Figures S13–S18).

### 2.6. Spectrofluorimetric Investigation of Compounds **3a**–**d**

Fluorescence excitation and emission spectra were recorded on a Shimadzu RF-20A (Shimadzu Corp., Kyoto, Japan) spectrofluorometric HPLC detector equipped with a Xenon lamp. This unit was attached to a Shimadzu CBM-20A communication module that enabled PC-controlled investigation through the Class-VP software (Shimadzu Corp., Kyoto, Japan). A 10 ng/mL acetonitrile sample solution was prepared for each measurement and added into the detector chamber through a capillary. Data were obtained by first recording the background spectrum of the solvent, followed by the analysis of the sample solution. The spectrum scanning option provided an automatic correction of spectral information with the previously recorded background. Before changing the sample or a mode of measurement, the detector capillary was washed multiple times with acetonitrile, and a new background value was consequently recorded. The emission spectra of derivatives were recorded between intervals of 500–750 nm using 488 nm as the excitation wavelength. The settings of the detector were as follows: “1X” detection gain, “high” sensitivity, and 120 nm/min scanning speed. The obtained background corrected emission spectra are shown in the Supporting Information, Figure S19.

### 2.7. Cell Lines

Human breast cancer (MDA-MB-231 cells and MCF-7 cells) cell lines were purchased from American Type Culture Collection (Rockville, MD, USA). These cell lines were cultured in MEM supplemented with 10% FBS, 1% non-essential amino acids and an antibiotic–antimycotic mixture (MTT assay, cell cycle analysis and caspase-3 assay), or Dulbecco’s Modified Eagle’s medium (DMEM) supplemented with 10% heat-inactivated FBS, 2 mM L-glutamine, 10,000 U/mL penicillin, and 10 mg/mL streptomycin (all other bioassays) at 37 °C in a humidified atmosphere containing 5% CO_2_. All cell lines were sub-cultured using 0.25% trypsin/EDTA. The density of cells for each experiment was determined with a Luna Automatic Cell Counter (South Korea) using a hemocytometer and trypan blue solution [34,35].

### 2.8. Cell Viability Assay

The colorimetric MTT ([3-(4,5-dimethylthiazol-2-yl)-2,5-diphenyltetrazolium bromide]) assay was used to estimate the ability of prepared compounds to inhibit the proliferation of human breast cancer cells (MCF-7 and MDA-MB-231), as previously described [36,37]. Briefly, the cells were seeded in 96-well microplates at a density of 5000 cells/well in a final volume of 100 µL and allowed to adhere overnight under standard conditions. Prior to testing, compounds were dissolved in DMSO as a 10 mmol/L stock solution and stored at −20 °C with minimal exposure to light to avoid oxidation. The stock solution was diluted with the culture medium before each treatment to obtain the final concentrations for the different experiments. Cells were exposed to 10 different concentrations of each compound (0.039, 0.07, 0.1, 0.31, 0.62, 1.25, 2.5, 5, 10, and 20 µM). Cisplatin was used as a positive control (0.1, 0.3, 0.6, 1.25, 2.5, 5, 10, and 20 µM). The plates were incubated for 72 h in the same conditions at 37 °C in a humidified atmosphere of 5% CO_2_. After incubation, 44 µL of the MTT solution (5 mg/mL PBS) was added to each well and incubated for 4 h. Subsequently, the supernatant was carefully removed, and insoluble purple formazan crystals were solubilized by adding 100 µL/well of DMSO and gently shaking them for 1 h at 37 °C (Stat Fax-2200, Awareness Technology INC, Palm City, FL, USA). Absorbance was measured at a wavelength of 545 nm using an automatic microplate reader (Stat Fax-2100; Awareness Technology INC, Palm City, FL, USA). Data were collected from two separate experiments performed in triplicate for each concentration and evaluated by Graph Pad Prism 5.01 (Graph Pad Software, San Diego, CA, USA). The half-maximal inhibitory concentration (IC_50_) values were calculated by the nonlinear regression model log inhibitor vs. normalized response.

### 2.9. Combination Study of Relevant Fragments Compared with Their Hybrids ***3b**–**d***

Two combination studies were performed: a virtual and an experimental one. In the case of the virtual study, cell viability data obtained following treatment with the hybrid compounds **3b**–**d** were analyzed in comparison with data obtained for their corresponding relevant fragments, such as compounds **1** and **8b**–**d**, and the hybrid compounds were considered as 1:1 ratio mixtures of compound **1** and the corresponding fragment. Two independent experiments were performed, each in triplicate. In the case of the experimental combination study, cell viability data obtained for equimolar mixtures of the abovementioned fragments were tested in comparison with the corresponding single-treatment controls. In this bioassay, two separate experiments were performed, each in duplicate.

Calculations were performed using the CalcuSyn software [38]. In each case, raw cell viability data from all replicates were averaged, and the resulting single dataset was analyzed as suggested by Chou [38]. CI values obtained this way were then used to mathematically describe the extent of pharmacological benefit gained by the coupling (virtual combination) in comparison with a combined effect of the mixture of fragments (experimental combination). “Synergism” in the virtual combination means that the hybrid exerts significantly stronger cytotoxicity than just a sum of that of its fragments.

### 2.10. Cell Death Analysis

The percentage of apoptotic, necrotic, and viable cells was determined by Annexin-V (AV)/PI labeling. Cells labeled with AV-FITC/PI were analyzed by flow cytometry. After 72 h treatments with 500 nM of each compound, both attached and floating cells of all samples (untreated and treated) were collected, washed in PBS to eliminate culture medium, and placed in 100 µL of binding buffer containing AV-FITC and PI in a ratio of 1:1 (*v*/*v*) (10 min at room temperature in the dark). Afterward, 400 µL of binding buffer was added to stop the reaction, and samples were analyzed within 1 h by flow cytometry. The fluorescence intensities of AV-FITC and PI were measured in the green (FL1) and red (FL2) channels on a CyFlow Space flow cytometer (Partec, Münster, Germany), respectively. In each sample, the fluorescence intensity of 20,000 cells was documented, while the amount of viable (AV−/PI−), early apoptotic (AV+/PI−), late apoptotic (AV+/PI+), and necrotic (AV−/PI+) cells was analyzed with Summit software (Dako Colorado Inc., USA). The experiments were performed in triplicate. For statistical analysis, two-way analysis of variance (ANOVA) with Dunnett’s multiple comparisons test was conducted using GraphPad Prism 6 software. Differences were considered statistically significant in comparison with untreated controls when *p* ≤ 0.05.

### 2.11. Cell Cycle Analysis in MDA-MB-231 Cells

To detect the cellular DNA content using PI staining, cell cycle distribution was determined by flow cytometry, as previously described [39]. Briefly, cells were seeded in six-well plates at a density of 4 × 10^5^ cells per well. On the second day, the MDA-MB-231 cells were treated with compounds at their IC_50_ or 2 × IC_50_ concentration. These concentrations were 0.2 and 0.4 µM (**3c**) or 0.3 and 0.6 µM (**3b**), respectively, while the control group was treated with MEM. Subsequently, cells were harvested with trypsin (250 µL/well), washed with PBS, resuspended and fixed with 70% EtOH, and kept at −20 °C. Directly before the assay, the fixed cells were washed with cold PBS and stained with PI in the presence of RNAse, Triton-X-100, and sodium citrate. Then, they were incubated in the dark at room temperature for 1 h. Finally, the DNA content was analyzed by flow cytometry (Partec CyFlow, Partec GmbH, Münster, Germany), with at least 20,000 cells being evaluated for each analysis. The experiment was performed in triplicate. Cell cycle distributions were determined by ModFit LT 3.3.11 software (Verity Software House, Topsham, ME, USA), and results are shown in the Supporting Information, Figure S20.

### 2.12. Effect of Compound ***3c*** on Caspase-3 Activity in MDA-MB-231 Cells

Caspase-3 activity was determined using a Caspase-3 Colorimetric Assay Kit, according to the manufacturer’s protocol, as previously published [40]. Briefly, cells were plated at the density of 12 × 10^6^ cells per 175 cm^2^ in a flask and allowed to attach and grow for 24 h. Then, they were treated with the appropriate concentrations of compound **3c** for 24 or 48 h, scraped, washed with PBS, and resuspended in Lysis Buffer. The supernatant was collected. Assays were performed in a 96-well plate by incubating 5 µL of the cell lysates in 100 µL of assay buffer containing 222 µM/L of the caspase-3 substrate at 37 °C in the dark for 24 h. Finally, the absorbance was measured at 405 nm with a microplate reader (Stat Fax 2100, Awareness Technology INC, Palm City, FL, USA). The comparison of the absorbance of the treated samples with the untreated controls to determine the change in caspase activity was performed by Graph Pad Prism 5.0 using a one-way analysis of variance (ANOVA) followed by Dunnett’s post-hoc test. Results are shown in the Supporting Information, Figure S21.

### 2.13. Effect of Compounds ***3a**–**d*** on DNA Damage Response

The effect of compounds **3a**–**d** on the ATR-dependent phosphorylation of Chk1 was evaluated by western immunoblotting, as previously published [16]. Briefly, MCF7 cells were pretreated with 1 µM of the compounds for 30 min and exposed to 10 µM cisplatin in the presence or absence of the compounds for 8 h to induce DDR. The same concentration of protoapigenone was used as a positive control for inhibition. The primary antibody against S345 of Chk1 was purchased from Cell Signaling Technology (Danvers, MA, USA); Chk1 and β-actin antibodies were purchased from Santa Cruz Biotechnology Inc (Dallas, TX, USA). The protein expression signal on blots was quantified by Fujifilm Multi Gauge software (Tokyo, Japan). The ratio of Chk1-S345 to Chk1 expression was calculated, and the means between the groups were compared by a one-way ANOVA. Data represent the mean ± standard deviation from three independent experiments; *: *p* < 0.05, **: *p* < 0.01.

### 2.14. Effect of Compounds ***3a**–**d*** on ROS/RNS Levels

DHE (life Technologies, D23107, NY, USA) and DHR (Sigma-Aldrich, D1054, MO, USA) fluorescent dyes were used to assess ROS and reactive nitrogen species (RNS) levels, respectively, in breast cancer cells. DHE fluorescence is activated by superoxide anion and corresponds to the intracellular ROS levels, while DHR fluorescence is activated by hydrogen peroxide and peroxynitrite anions and corresponds to the intracellular RNS levels. MCF-7 and MDA-MB-231 cells were plated and incubated overnight in 6-well plates at a density of 100,000 cells/well. After a 24 h incubation with 1 µM of each compound, adherent cells were harvested by trypsinization and placed in medium containing 5 µM of DHE or DHR for 30 min at 37 °C in the dark. Afterward, cells were washed twice in PBS. DHE and DHR fluorescence were assessed in the FL2 red channel and FL1 green channel, respectively. Data from a minimum of 20,000 cells were collected and assayed for each sample. These experiments were repeated three times. The samples were analyzed on a CyFlow Space flow cytometer (Partec, Münster, Germany). A two-way ANOVA with Dunnett’s multiple comparisons test was performed using GraphPad Prism 6 software. Differences were considered statistically significant in the comparison with untreated controls when *p* ≤ 0.05.

### 2.15. Assessing Mitochondrial Membrane Depolarization

JC-1 (BD Biosciences, San Diego, USA) is a cationic dye that can provide information about the mitochondrial membrane potential. JC-1 accumulates in healthy mitochondria as red fluorescent aggregates, while in depolarized dysfunctional mitochondria, JC-1 remains in the cytoplasm as green fluorescent monomers. During the depolarization of mitochondria, JC-1 labeled monomers leak out of the mitochondria into the cytoplasm, increasing the ratio of green to red fluorescence. MCF-7 and MDA-MB-231 cells were incubated overnight in 6-well plates (100,000 cells/well), and then treated for 24 h with 1 µM of each compound. Subsequently, the cells were incubated with the JC-1 reagent for 15 min at 37 °C. After two washing steps in 1X Assay Buffer, the cells were resuspended in PBS. Both red and green fluorescence emissions were detected, and their ratio was analyzed on a CyFlow Space flow cytometer (Partec, Münster, Germany). Data from a minimum of 20,000 cells were collected per sample. All experiments were performed in triplicate. A two-way ANOVA with Dunnett’s multiple comparisons test was applied using GraphPad Prism 6 software. Differences were considered statistically significant in comparison with untreated controls when *p* ≤ 0.05.

## 3. Results and Discussion

### 3.1. Chemistry

**Scheme 1:**
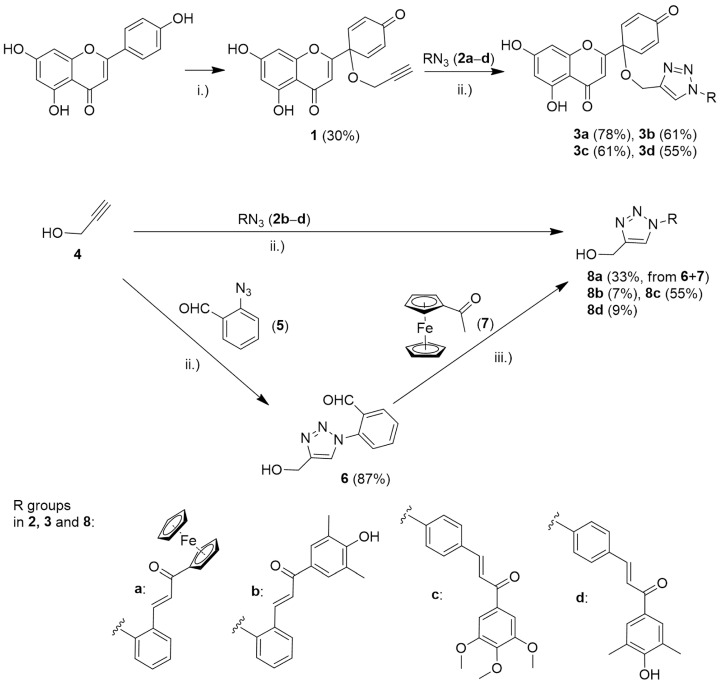
Synthesis of the tested protoflavone-chalcone hybrids with triazole linkers (**3a**–**d**) and the corresponding hydroxymethyl derivatives (**8a**–**d**) serving as reference compounds for evaluating the pharmacological benefit gained by the coupling (see Figure 1). Reaction conditions: (i) PIFA (2.0 eq.), acetonitrile:propargyl alcohol (9:1), 80 °C, 1h; (ii) azide (1.0 eq.), alkyne (1.0 eq.), CuSO_4_ (0.2 eq.), sodium ascorbate (1.0 eq.), *n*-BuOH:water (1:1), rt, 12 h; (iii) **6** (1.0 eq.), **7** (1.0 eq.), NaOH, water, EtOH, room temperature, 12 h.

The protoflavone structure of protoapigenone 1′-*O*-propargyl ether (compound **1**) was prepared by a hypervalent iodine-induced oxidative de-aromatization from apigenin, using bis(trifluoroacetoxyiodo)benzene (PIFA) as previously published [33]. The terminal alkyne fragment of this compound was used for linking it to a preliminary selection of chalcone-based azide components **2a**–**d** by copper(I)-catalyzed click reactions [41] conducted under well-established ascorbate-conditions to generate the targeted protoflavone-chalcone hybrids with 1,4-disubstituted 1,2,3-triazole linkers (**3a**–**d**) in acceptable to good yields (Scheme 1). The synthesis of hydroxymethyl derivatives **8a**–**d**, serving as reference compounds for the combination studies, was also attempted under the same conditions using propargyl alcohol **4** as an alkyne (Scheme 1). While the conversion using **2a** as an azide component was not successful, and the reactions with azides **2b** and **2d** containing a 4-hydroxy-3,5-dimethylphenyl group produced **8b** and **8d** in low yields (7% and 9%, respectively), the cyclization of **2c** containing a 3,4,5-trimethoxyphenyl group resulted in the formation of **8c** in an average yield. The ferrocene analog **8a** was prepared by a two-step procedure, starting with the facile copper(I)-mediated cyclization of **4** and 2-azidobenzaldehyde **5** followed by the base-catalyzed condensation of the resulting triazole (**6**) with acetylferrocene (**7**).

The compounds’ structures were confirmed by high-resolution mass spectrometry (HRMS) and nuclear magnetic resonance (NMR) spectroscopy. The measured ^1^H and ^13^C NMR data of the novel compounds, including hybrids **3a**–**d** (see Supporting Information, Figures S1–S8), are consistent with their structure. However, the following remarks are necessary to make: (i) in **3a**–**d,** the connection of the protoflavone and chalcone fragments was unambiguously evidenced by the cross peaks discernible in the HMBC spectra generated by a three-bond interaction between the isotropic H15 protons in the OCH_2_ group and the sp^3^ quaternary C9 carbon in the cycloxehadienone ring; (ii) the singlet signal of H15 protons is also correlated with the ^13^C NMR signals of the C16 and C17 triazole carbons generated by two-bond and three-bond interactions, respectively; (iii) an HMBC correlation was detected between H3 and C9 transmitted by a three-bond coupling; (iv) the *E*-configuration of the C=C double bond is reflected from the coupling constant of ca. 15 Hz characterizing the interaction involving the protons in the enone moiety. Complete NMR characterization and ^1^H and ^13^C NMR spectra of compounds **3a**–**d**, **6**, and **8a**–**d** are available in the Supporting Information (Figures S1–S18).

Spectrofluorimetric investigations were used to investigate the possibility of spectral interference related to the compounds’ native fluorescence, considering the high degree of aromaticity of the hybrid compounds **3a**–**d** and the several fluorescence-based bioassays that we planned to perform on them (see below). Emission spectra were recorded between intervals of 500–750 nm using 488 nm as the excitation wavelength, and they are available in Supporting Information (Figure S19). None of these derivatives showed significant fluorescent emission under these experimental circumstances. Therefore, their fluorescent interference during the flow cytometry analyses was not expected.

### 3.2. Cell Viability Assay, Virtual and Experimental Combination Study

We evaluated the cytotoxic activity of relevant fragments and their hybrids (**3a–d**) on breast cancer cell lines by MTT assay. For the selection of relevant fragments, the following points were considered: (i) the *p*-quinol protoflavone containing a free OH group at position C-1′ (i.e., protoapigenone in our case) is generally more toxic than its 1′-*O*-alkyl derivatives, and all hybrid molecules have an alkoxy-function at this position; and (ii) the free azide of compounds **2a**–**d** makes them potentially toxic in a way that does not have relevance in view of the hybrids. Therefore, for each hybrid compound of type **3**, protoapigenone 1′-*O*-propargyl ether (**1**) and the corresponding chalcone coupled to hydroxymethyltriazole (**8a**–**d**) were selected as reference fragments. Comparative testing of the cytotoxicity of these compounds provides a good assessment of the pharmacological benefit gained by the coupling, despite the slight overlap between the chemical structure of these fragments meaning a (necessary) compromise, as compared to evaluating the two halves of each hybrid molecule.

The MCF-7 cell line was used as a representative model for estrogen receptor (ER)-positive breast cancer, while the MDA-MB-231 cell line was used as a model of triple-negative breast cancer (TNBC). TNBCs are highly resistant to first-line antiestrogen therapy, and therefore their treatment relies on the use of chemotherapeutics. Both cell lines have an epithelial phenotype, although a mesenchymal marker, vimentin, was determined in the MDA-MB-231 cell line [42]. Both cell lines are tumorigenic and adherent while they differ in their tissue spatial organization. MCF-7 is a luminal subtype A human breast cell line [43], and MDA-MB-231 is a basal breast cell line originating from a metastatic site [44]. The cells were treated with each compound in the concentration range between 30 nM and 20 µM for 72 h; results are presented in Table 1.

All hybrid compounds showed a potent, dose-dependent cytotoxic effect against the tested cancer cell lines. Except for **3b**, the compounds were also slightly more effective against the TNBC cell line MDA-MB-231.

When comparing the effect of the hybrids to their fragments, compound **3a** was found to be ca. 3.7 times more potent on MCF-7 cells and ca. 6.8 times more potent on MDA-MB-231 cells than compound **1**. This demonstrates a strong potentiation of the cytotoxic effect when adding the non-cytotoxic fragment **8a** to the structure of the hybrid.

**Figure 1:**
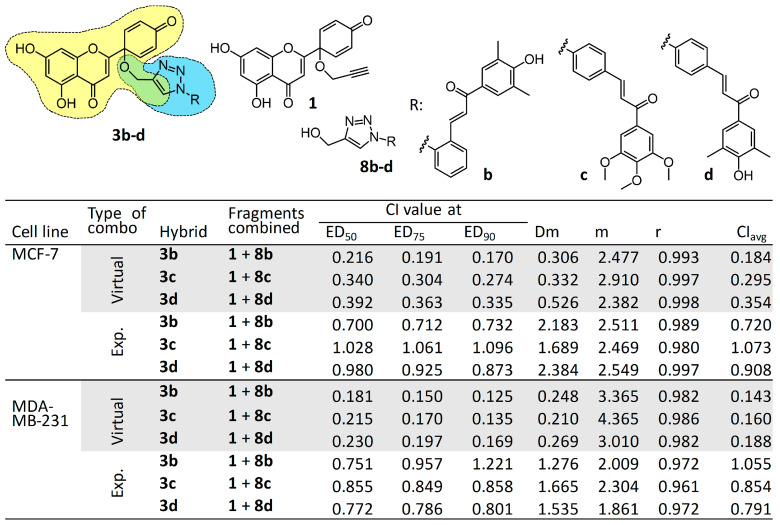
Comparative analysis of the cytotoxic activity of the hybrid compounds **3b–d** by the Chou-Talalay method with that of their fragments. Results of a virtual (hybrid vs. fragments alone) and an experimental (Exp.: equimolar mixture of fragments vs. fragments alone) combination study are shown at 50%, 75%, and 90% of inhibition. CI: combination index; 0 < CI < 1, CI = 1, and CI > 1 represent synergism, additivity, and antagonism, respectively. Dm, m, and r represent the antilog of the x-intercept (activity), slope (shape of the dose-effect curve), and linear correlation coefficient (conformity of the data) of the median effect plot, respectively [38]. CI_avg_ = (CI_50_ + 2 × CI_75_ + 3 × CI_90_)/6. The lowest CI_avg_ value demonstrates the highest added benefit of hybridization in terms of in vitro cytotoxic activity.

However, in the case of compounds **3b**–**d**, each fragment (**1** and **8b**–**d**, respectively) exhibited significant cytotoxic effects. Therefore, we performed a more sophisticated comparative evaluation to quantitatively assess the added pharmacological benefit of linking each pair of fragments into a hybrid molecule. To the end of performing this evaluation, we selected the Chou–Talalay method, which offers a well-established mathematical model for the calculation of drug–drug interactions [38]. Two separate analyses were performed. On one hand, the method was used in an unusual way that may be referred to as a “virtual combination study”. Treatment with the hybrid molecules **3b**–**d** was considered as a 1:1 ratio combination treatment with their two relevant corresponding fragments (i.e., **1** and **8b**, **1** and **8c**, and **1** and **8d**, respectively), and the cell viability data presented in Table 1 were re-evaluated accordingly using the CalcuSyn software. Furthermore, a classical experimental combination study was also performed as a control, in which equimolar combinations of compounds **1** and **8b**, **8c**, or **8d** were analyzed in comparison with the corresponding single treatments. The results of these calculations are presented in Figure 1.

The strong synergism observed by the virtual combination study for nearly every hybrid clearly showed that these compounds were much more potent than what would be expected by their corresponding building blocks. It is also evident that the co-treatment of cells with the fragments (see Exp. rows in the table of Figure 1) resulted in additive effects or moderate synergism at best. Our results therefore strongly suggest that the hybridization of these fragments offers a relevant pharmacological benefit.

Using the Chou–Talalay method as a mathematical tool to perform a quantitative comparison between the bioactivity of two fragments and their corresponding hybrid is, to the best of our knowledge, a novel approach. We believe that with an appropriate selection of fragments to evaluate, such a virtual combination study provides a reasonable and easy-to-use platform to assess the bioactivity of hybrid compounds in general, therefore, we suggest an extension for the applicability of the Chou–Talalay method to analyze related bioactivity data.

### 3.3. Cell Death Induction Analysis

**Figure 2:**
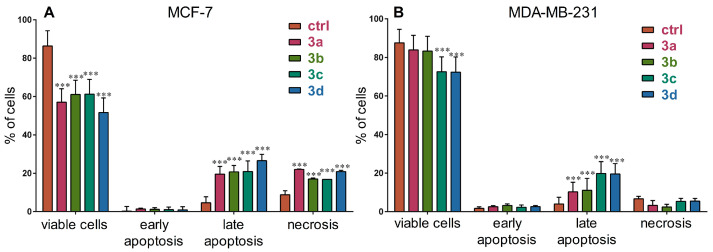
Cell death induction by the hybrid compounds in MCF-7 (**A**) and MDA-MB-231 cells (**B**). Cells were treated with 500 nM of each compound for 72 h, ***: *p* < 0.001 by two-way analysis of variance (ANOVA) followed by Dunnett’s multiple comparisons test. Necrosis refers to primary necrosis.

To further investigate the effects of the hybrid compounds on the two breast cancer cell lines, cell death induction by compounds **3a**–**d** was analyzed (Figure 2).

While a substantial increase in primary necrosis was present only in MCF-7 cells, significant induction of late apoptosis was observed after all treatments in both cell lines. The viability of MCF-7 cells was significantly decreased by all of the compounds, whereas only **3c** and **3d** decreased the viability of MDA-MB-231 cells after 72 h. Of note, MCF-7 cells also responded differently to the treatment than MDA-MB-231 cells. Specifically, more necrosis (primary necrosis) was observed in MCF-7 cells. Cell type-dependent occurrence of either apoptotic or necrotic cell fate was previously observed for protoapigenone, and it was suggested that this may be connected to its effect on the DDR [16].

### 3.4. Cell Cycle Analysis and Effects of Compound ***3c*** on Caspase-3 Activity in MDA-MB-231 Cells

Compound **3c** was also tested for its effect on the cell cycle and caspase-3 activity in this cell line, because its potent cytotoxic activity was the strongest against MDA-MB-231 cells. Cells were treated with IC_50_ or double IC_50_ concentrations of **3c**. The results of these bioassays are presented in Supporting Information (Figures S20 and S21). As a summary, it was concluded that, in comparison with the control groups, the treatment significantly increased the hypodiploid (subG1) phase, and this was associated with a significant decrease in the G1 phase in a dose-dependent manner after a 24 h treatment. These results (subG1 portion) indicate apoptotic cell death and that this hybrid compound inhibits DNA synthesis in a dose-dependent manner, which is consistent with previous results published for protoapigenone [45]. However, unlike protoapigenone [45], **3c** did not produce any significant effects on the proportion of cells in S and G2/M phases at the tested concentrations. Compound **3c** also caused a time- and concentration-dependent increase in caspase-3 activity, which is in agreement with previous reports on the pro-apoptotic activity of protoapigenone [12,17].

### 3.5. Effects of Compounds ***3a**–**d*** on the ATR-Dependent Phosphorylation of Chk1

**Figure 3:**
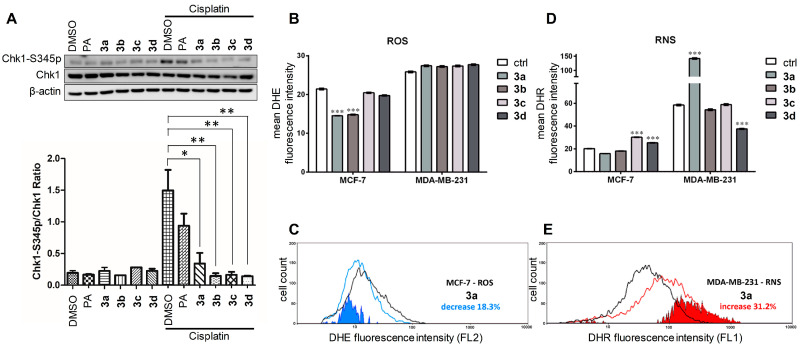
Effect of compounds **3a**–**d** on the DNA damage response and redox balance in breast cancer cells. (**A**) Effect of compounds **3a**–**d** on Chk1 phosphorylation in MCF-7 cells, PA: protoapigenone; (**B**) Changes in reactive oxygen species (ROS) production induced by 1 µM of compounds **3a**–**d** in MCF-7 and MDA-MB-231 cells assessed after 24 h; (**C**) Decrease in ROS production by **3a**; (**D**) Changes in reactive nitrogen species (RNS) production induced by 1 µM of compounds **3a**–**d** in MCF-7 and MDA-MB-231 cells assessed after 24 h; (**E**) Increase in RNS production by **3a**; ctrl: untreated cell control.

It was of primary interest to study whether the hybrid compounds retained activity on this target due to the bioactivity of the key fragment protoapigenone on the ATR-dependent inhibition of Chk1 in the DDR. Therefore, the effects of compounds **3a**–**d** on the cisplatin-induced activation of Chk1 were determined (Figure 3A). In comparison with the positive controls treated with protoapigenone, compounds **3a**–**d** significantly decreased and almost abolished cisplatin-induced Chk1-S345 phosphorylation. Checkpoint kinases play a critical role in the DDR to maintain genome integrity by mediating DNA repair or cell apoptosis. Agents that inhibit ATR or Chk1 have been developed in preclinical and clinical studies to improve tumor sensitivity by inducing tumor death in the presence or absence of DNA damaging therapies, such as radiotherapy and chemotherapy, or to exploit synthetic lethality interactions between Chk1 or ATR inhibition and genetic defects in other DDR genes [20,21]. Supporting the strong synergism identified in the virtual combination study, the hybrid compounds were more potent ATR inhibitors than their protoflavone fragment.

It is of interest that the expression levels of Chk1 and the capacity for DNA repair were most recently found to be associated with cancer sensitivity to DNA damaging agents, such as mitomycin C (MMC). In contrast with MMC-sensitive MCF-7 cells, MDA-MB-231 cells are resistant to MMC. This relates to the fact that MDA-MB-231 cells express high levels of Chk1 protein as well as MMC-induced Chk1-p and have a low DNA repair capacity [46]. This relative resistance of MDA-MB-231 cells to DNA damaging agents was also seen in our current study from their poor response to cisplatin treatment: we found them ca. five times more resistant to cisplatin as compared with MCF-7 cells. Cancer cells with higher Chk1 expression would expectably be sensitive to Chk1 inhibition. Further, it is known that the MDA-MB-231 cell line is a p53 mutant [47], in contrast with MCF-7 that contains wild-type p53 [48], and p53 mutations are among the factors that predict vulnerability of cancer cells to ATR inhibitors [49]. Taken together, it seems likely that potent inhibition of the ATR-dependent activation of Chk1 by compounds **3a**–**d** is among the reasons for their strong cytotoxicity on the TNBC cell line MDA-MB-231.

### 3.6. Effect on the Levels of Reactive Oxygen and Nitrogen Species

Based on the pro-oxidant properties of both protoflavone and chalcone, their effects on intracellular ROS and/or reactive nitrogen species (RNS) levels were studied (Figure 3B–E).

Although the results cannot point to a uniform pattern of action, such as antioxidant or pro-oxidant activity, all of the compounds demonstrated the ability to interfere with the redox system in breast cancer cells. Regarding ROS levels, **3a** and **3b** significantly decreased superoxide anion levels in MCF-7 cells. Additionally, **3c** and **3d** significantly increased RNS levels in MCF-7 cells. The most prominent effect on RNS levels was observed with **3a,** suggesting its significant pro-oxidant activity in MDA-MB-231 cells. In contrast, **3d** significantly decreased the RNS levels in MDA-MB-231 cells.

The changes of ROS and RNS after treatment with the hybrid compounds can be both time- and cell line-dependent. The end-point detection after 24 h showed that the cellular antioxidant capacity is modified by all tested compounds. Interestingly, according to our results, untreated MDA-MB-231 cells show increased production of ROS and RNS levels as compared with untreated MCF-7 cells (Figure 3B–E), indicating that this TNBC cell line has a higher oxidative status. Indeed, previous findings showed that in comparison with MCF-7 cells, MDA-MB-231 cells have higher FAD/NADPH redox ratio that is an indicator of oxidized mitochondrial state [50]. MCF-7 cells depend on energy produced from oxidative phosphorylation in normoxic conditions but could switch to glycolytic activity under hypoxia, while MDA-MB-231 cells predominantly use glycolysis regardless of the availability of oxygen [51]. In addition, MCF-7 cells possess high levels of glutathione peroxidase, while MDA-MB-231 cells contain increased levels of glutathione transferase [52]. Therefore, high oxygen consumption of MCF-7 cells requires more efficient antioxidant system than that of MDA-MB-231 cells that do not depend on oxygen. Consequently, MDA-MB-231 cells may be more vulnerable to pro-oxidants than MCF-7 cells. This may explain obvious discrepancy in the effect of **3a**, a compound with a prominent antioxidant effect in MCF-7 cells as seen from the decrease in ROS levels (Figure 3B,C), and with a prominent pro-oxidative effect in MDA-MB-231 cells as seen from the increase in RNS levels (Figure 3D,E). Similar opposite effects on the ROS and RNS production were previously observed with ferrocene–quinidine hybrids [29] containing the same triazole-linked ferrocene moiety as compound **3a**.

It is worth mentioning that the administration of pro-oxidants to exploit oxidative stress-related vulnerabilities of cancer may also lead to unwanted toxicity in healthy cells and tissues. Therefore, it will be particularly important to conduct future studies elaborating related properties of our hybrid compounds and/or to assure their adequate selective targeting at the tumor site.

### 3.7. Effect on Mitochondrial Membrane Depolarization

**Figure 4:**
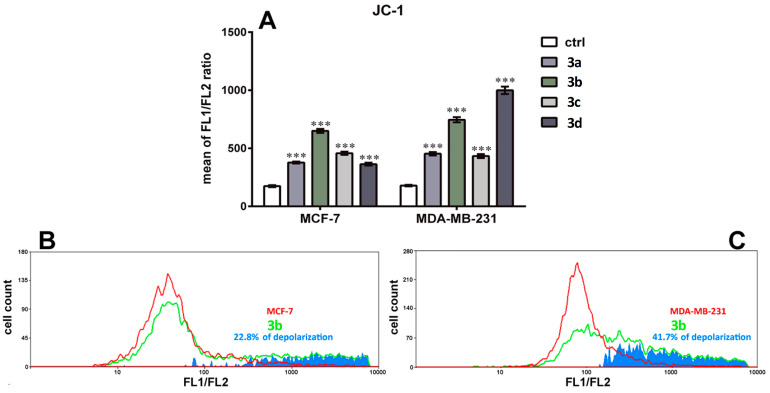
Hybrid-protoflavones induce mitochondrial membrane depolarization. (**A**) Increase in the ratio of green to red fluorescence (FL1/FL2) assessed by JC-1 staining in MCF-7 and MDA-MB-231 cells after 24 h treatments with 1 µM of compounds **3a**–**d**; (**B**) Illustration of the effect induced by **3b** in MCF-7 cells; (**C**) Illustration of the effect induced by **3b** in MDA-MB-231 cells.

Activating the mitochondrial apoptosis pathway, chalcones are commonly associated with the ability to interfere with the mitochondrial electron chain [53,54], and changes in the ROS and RNS levels may also affect mitochondrial function. Therefore, we determined the ability of hybrid compounds **3a–d** to change the mitochondrial membrane potential in MCF-7 and MDA-MB-231 breast cancer cells (Figure 4).

All of the compounds induced significant depolarization of the mitochondrial membrane in both cell lines. While the effects of compounds **3a**–**c** were similar in MCF-7 and MDA-MB-231 cells, compound **3d** showed a >2-fold increase in selectivity toward MDA-MB-231 cells in this regard. While this is just one aspect of a clearly multitarget antitumor action of these compounds, it may still be worth noting that **3d** also showed the highest selectivity toward MDA-MB-231 cells vs. MCF-7 cells among the hybrids in the cytotoxicity assay (see Table 1). In addition, the induction of late apoptosis after 72 h treatment by **3d** and other hybrid compounds (Figure 2) is probably due to pro-apoptotic factors released from affected mitochondria whose proton leakage was observed after 24 h treatments.

## 4. Conclusions

The hybrid compounds prepared in this study exhibited a significant increase in their antitumor activity compared with either fragment alone, resulting in potent antitumor leads active in the sub-micromolar concentration range against breast cancer cell lines. The strong activity of these compounds against the TNBC model is particularly promising since the treatment of this disease is challenging due to its resistance to first-line therapies. In this respect, compound **3a** deserves to be further studied as a pro-oxidant, as well as compound **3d**, which exerted the most pronounced effect on mitochondrial depolarization.

The triazole coupling of the ATR inhibitor protoflavone and a chalcone fragment resulted in hybrids that clearly act on multiple targets. They exert a significantly stronger activity on the cisplatin-induced activation of Chk1 as compared with their parent fragment protoapigenone, interfere with the redox balance of cells, and strongly depolarize the mitochondrial membrane. The potency and multitarget antitumor action of these hybrid compounds make them valuable starting points for possible further developments.

## Figures and Tables

**Table 1:** Cytotoxic activity of hybrid compounds **3a**–**d** and their corresponding fragments **1** and **8a**–**d** on human breast cancer cell lines. Confidence interval: 95% CI, *n* = 6 from two biological replicates (*n* = 3 each). Positive control: cisplatin, *n* = 10 from two biological replicates (*n* = 5 each).

	IC_50_ [95% CI] (µM)	
Compound	MCF-7	MDA-MB-231
**1**	1.74 [1.55–1.95]	2.53 [2.34–2.72]
**8a** ^a^	>20	>20
**8b**	15.07 [13.66–16.64]	11.11 [10.53–11.71]
**8c**	2.51 [2.24–2.82]	4.40 [3.98–4.86]
**8d**	11.00 [10.20–11.85]	4.92 [4.52–5.36]
**3a**	0.47 [0.45–0.49]	0.37 [0.36–0.39]
**3b**	0.25 [0.23–0.28]	0.29 [0.27–0.31]
**3c**	0.30 [0.27–0.32]	0.22 [0.21–0.24]
**3d**	0.51 [0.48–0.55]	0.32 [0.30–0.35]
Cisplatin	5.35 [4.97–5.76]	26.15 ^b^ [24.18–28.27]

^a^**8a** exhibited less than 10% inhibition on each cell line at the highest tested dose of 20 µM; ^b^ Experimental data are available up to 20 µM, IC_50_ value was extrapolated from the nonlinear curve fitting (log inhibitor vs. normalized response model) by GraphPad Prism 5.0.

## Supplementary Materials

The following are available online at https://www.mdpi.com/2076-3921/9/6/519/s1, Complete NMR characterization, HRMS data, and the ^1^H and ^13^C NMR spectra of compounds **3a**–**d**, **6**, and **8a**–**d** (Figures S1–S18), fluorescence emission spectra of compounds **3a**–**d** (Figure S19), bioactivity results for compounds **3b** and **3c** on the cell cycle of MDA-MB-231 cells (Figure S20), and for compound **3c** on the caspase-3 activity of MDA-MB-231 cells (Figure S21).
