# Supplementary material for: Protoflavone-Chalcone Hybrids Exhibit Enhanced Antitumor Action through Modulating Redox Balance, Depolarizing the Mitochondrial Membrane, and Inhibiting ATR-Dependent Signaling
Source: Antioxidants (Basel). 2020 Jun 12;9(6):519. doi: 10.3390/antiox9060519 (PMC7346169; doi:10.3390/antiox9060519)

Supporting Information
for

Protoflavone-Chalcone Hybrids Exert Enhanced Antitumor Action Through Modulating Redox Balance, Depolarizing Mitochondrial Membrane and Inhibiting ATR-Dependent Signaling

Ahmed Dhahir Latif, Tamás Jernei, Ana Podolski-Renić, Ching-Ying Kuo, Máté Vágvölgyi, Gábor Girst, István Zupkó, Sedef Develi, Engin Ulukaya, Hui-Chun Wang, Milica Pešić, Antal Csámpai, Attila Hunyadi

Complete ^{1}H- and ^{13}C-NMR characterization of the prepared novel compounds 3
Compound **3a** 3
Compound **3b** 4
Compound **3c** 5
Compound **3d** 6
Compound **6** 7
Compound **8a** 7
Compound **8b** 8
Compound **8c** 8
Compound **8d** 9
^{1}H-NMR and ^{13}C-NMR spectra of the novel prepared compounds 10
Figure S1. ^{1}H-NMR spectrum of **3a** 10
Figure S2. ^{13}C-NMR spectrum of **3a** 10
Figure S3. ^{1}H-NMR spectra of **3b** 11
Figure S4. ^{13}C-NMR spectra of **3b** 11
Figure S5. ^{1}H-NMR spectra of **3c** 12
Figure S6. ^{13}C-NMR spectra of **3c** 12
Figure S7. ^{1}H-NMR spectra of **3d** 13
Figure S8. ^{13}C-NMR spectra of **3d** 13
Figure S9. ^{1}H-NMR spectra of **6** 14
Figure S10. ^{13}C-NMR spectra of **6** 14
Figure S11. ^{1}H-NMR spectra of **8a** 15
Figure S12. ^{13}C-NMR spectra of **8a** 15
Figure S13. ^{1}H-NMR spectra of **8b** 16
Figure S14. ^{13}C-NMR spectra of **8b** 16

Figure S15. ^{1}H-NMR spectra of **8c** 17

Figure S16. ^{13}C-NMR spectra of **8c** 17

Figure S17. ^{1}H-NMR spectra of **8d** 18

Figure S18. ^{13}C-NMR spectra of **8d** 18

Figure S19. Background corrected fluorescent emission spectra of compounds **3a-d** obtained upon exciting molecules at 488 nm. 19

Figure S20. Effect of compounds **3b** and **3c** on the cell cycle distribution of MDA-MB-231 cells. 20

Figure S21. Effect of compound **3c** on the caspase-3 activity of MDA-MB-231 cells. 20

Complete ^{1}H- and ^{13}C-NMR characterization of the prepared novel compounds

For each compound characterized in this session, the atomic numbering used for assignment of ^{1}H- and ^{13}C NMR signals do not correspond to IUPAC rules reflected from the given systematic names. For the sake of clarity, structures are also presented along with the numbering used for the NMR signal listing.

Compound 3a

(*E*)-2-(1-((1-(2-(3-ferrocenyl-3-oxoprop-1-en-1-yl)phenyl)-1*H*-1,2,3-triazol-4-yl)methoxy)-4-oxocyclohexa-2,5-dien-1-yl)-5,7-dihydroxy-4H-chromen-4-one (**3a**)

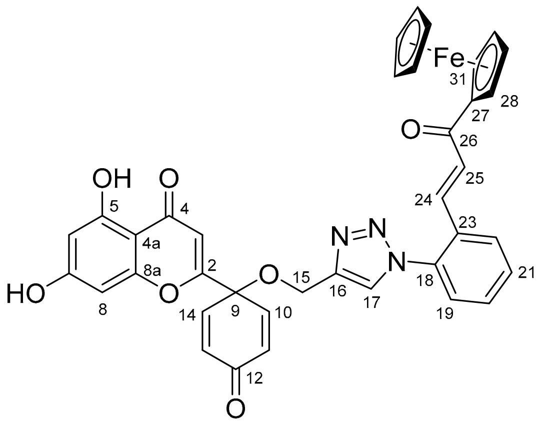

Purple solid; Yield: 165 mg (78%); ^{1}H-NMR (DMSO-d$_6$): 12.48 (s, 1H, C5O<u>H</u>), 10.95 (br s, 1H, C7O<u>H</u>), 8.68 (s, 1H, H17), 8.28 (br s, 1H, H22), 7.58 (br s, 1H, H19), 7.64-7.67 (m, 2H, H20 and H21), 7.34 (d, J = 15.3 Hz, 1H, H25), 7.19 (m, 3H, H10 and H14 and H24), 6.58 (m, 3H, H3 and H11 and H13), 6.22 (s, 1H, H8), 6.18 (s, 1H, H6), 4.96 (br s, 2H, H28 and H31), 4.80 (s, 2H, H15), 4.65 (br s, 2H, H29 and H30), 4.19 (s, 5H, η^5-C$_5$<u>H$_5$</u>); ^{13}C-NMR (DMSO-d$_6$): 192.1 (C26), 184.7 (C13), 182.0 (C4), 165.2 (C7), 164.6 (C2), 161.9 (C5), 157.8 (C8a), 145.8 (C10 and C14), 144.5 (C16), 136.6 (C18), 133.7 (C24), 132.8 (C11 and C13), 131.3 (C20 and C21), 130.9 (C23), 128.7 (C22), 127.5 (C19), 127.3 (C17), 127.2 (C25), 108.0 (C3), 104.4 (C4a), 99.7 (C6), 94.4 (C8), 80.7 (C27), 74.9 (C9), 73.5 (C29 and C30), 70.4 (η^5-<u>C$_5$</u>H$_5$), 70.1 (C28 and C31), 59.1 (C15). HRMS: $C_{37}H_{27}FeN_3O_7$, calculated m/z [M+H$^+$]: 682.12712, found: 682.12767.

Compound **3b**

(*E*)-5,7-dihydroxy-2-(1-((1-(2-(3-(4-hydroxy-3,5-dimethylphenyl)-3-oxoprop-1-en-1-yl)phenyl)-1*H*-1,2,3-triazol-4-yl)methoxy)-4-oxocyclohexa-2,5-dien-1-yl)-4*H*-chromen-4-one **(3b)**

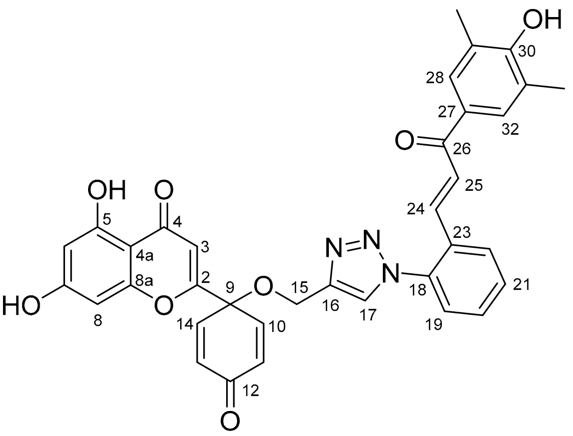

Yellow solid; Yield: 116 mg (61%); ^{1}H-NMR (DMSO-d_6): 12.46 (s, 1H, C5OH), 10.87 (br s, 1H, C7OH), 9.21 (br s, C30OCH$_3$), 8.63 (s, 1H, H17), 8.30 (dd, *J* = 7.8 Hz, 1.6 Hz, 1H, H22), 7.86 (d, *J* = 15.6 Hz, 1H, H25), 7.72 (s, 2H, H28 and H32), 7.67 (td, *J* = 7.6 Hz, 1.6 Hz, 1H, H20), 7.63 (td, *J* = 7.5 Hz, 1.1 Hz, 1H, H21), 7.55 (dd, *J* = 7.6 Hz, 1.1 Hz, 1H, H19), 7.19 (d, *J* = 9.9 Hz, 2H, H10 and H14), 7.18 (d, *J* = 15.6 Hz, 1H, H24), 6.55 (s, 1H, H3), 6.55 (d, *J* = 9.9 Hz, 2H, H11 and H13), 6.19 (d, *J* = 2.0 Hz, 1H, H8), 6.16 (d, *J* = 2.0 Hz, 1H, H6), 4.76 (s, 2H, H15), 2.19 (s, 6H, CH$_3$); ^{13}C-NMR (DMSO-d_6): 187.4 (C26), 184.7 (C13), 182.0 (C4), 165.2 (C7), 164.6 (C2), 161.9 (C5), 159.0 (C30), 157.8 (C8a), 145.8 (C10 and C14), 144.5 (C16), 136.7 (C18), 136.2 (C24), 132.8 (C11 and C13), 131.4 (C21), 131.2 (C23), 130.8 (C20), 130.2 (C28 and C32), 128.6 (C22), 127.4 (C17 and C19 and C27), 125.8 (C25), 124.7 (C29 and C31), 108.0 (C3), 104.4 (C4a), 99.7 (C6), 94.4 (C8), 59.1 (C15), CH$_3$). HRMS: $C_{35}H_{27}N_3O_8$, calculated m/z [M+H$^+$]: 618.18764, found: 618.18594.

Compound 3c

(*E*)-5,7-dihydroxy-2-(4-oxo-1-((1-(4-(3-oxo-3-(3,4,5-trimethoxyphenyl)prop-1-en-1-yl)phenyl)-1*H*-1,2,3-triazol-4-yl)methoxy)cyclohexa-2,5-dien-1-yl)-4*H*-chromen-4-one (**3c**)

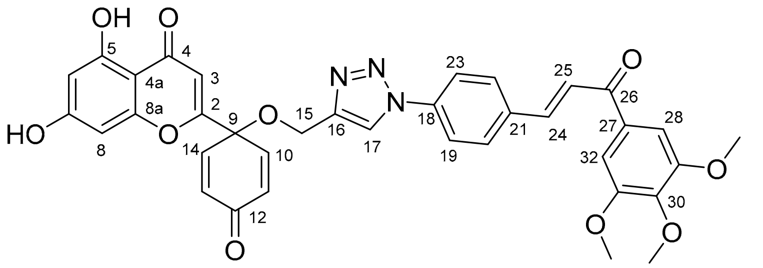

Yellow solid; Yield: 124 g (61%); ^{1}H-NMR (DMSO-d$_6$): 12.44 (s, 1H, C5OH), 10.92 (br s, 1H, C7OH), 8.97 (s, 1H, H17), 8.11 (d, *J* = 8.4 Hz, 2H, H20 and H22), 8.00 (d, *J* = 15.6 Hz, 1H, H25, overlapped by H19 and H23), 7.99 (d, *J* = 8.3 Hz, 2H, H19 and H23, overlapped by H25), 7.77 (d, *J* = 15.6 Hz, 1H, H24), 7.41 (s, 2H, H28 and H32), 7.15 (d, *J* = 9.9 Hz, 2H, H10 and H14), 6.56 (d, *J* = 9.9 Hz, 2H, H11 and H13), 6.53 (s, 1H, H3), 6.18 (s, 1H, H8), 6.15 (d *J* = 1.5 Hz, 1H, H6), 4.71 (s, 2H, H15), 3,87 (s, 6H, C29OCH_3 and C31OCH_3), 3.73 (s, 3H, C30OCH_3); ^{13}C-NMR (DMSO-d$_6$): 188.3 (C26), 184.7 (C12), 182.0 (C4), 165.2 (C7), 164.7 (C2), 161.9 (C5), 157.8 (C8a), 153.4 (C29 and C31), 145.7 (C10 and C14), 145.2 (C16), 142,8 (C24), 142.7 (C30), 138.0 (C18), 135,5 (C21), 133.3 (C27), 132.8 (C11 and C13), 130.9 (C20 and C22), 123.5 (C25), 123.1 (C17), 120.7 (C19 and C23), 108.0 (C3), 106.7 (C28 and C32), 104.4 (C4a), 99.7 (C6), 94.4 (C8), 74.9 (C9), 60.7 (C30OCH$_3$), 59.1 (C15), 56.8 (C29OCH$_3$ and C31OCH$_3$). HRMS: $C_{36}H_{29}N_3O_{10}$, calculated m/z [M+H$^+$]: 664.19082, found: 664.19312.

Compound 3d

(*E*)-5,7-dihydroxy-2-(1-((1-(4-(3-(4-hydroxy-3,5-dimethylphenyl)-3-oxoprop-1-en-1-yl)phenyl)-1*H*-1,2,3-triazol-4-yl)methoxy)-4-oxocyclohexa-2,5-dien-1-yl)-4*H*-chromen-4-one **(3d)**

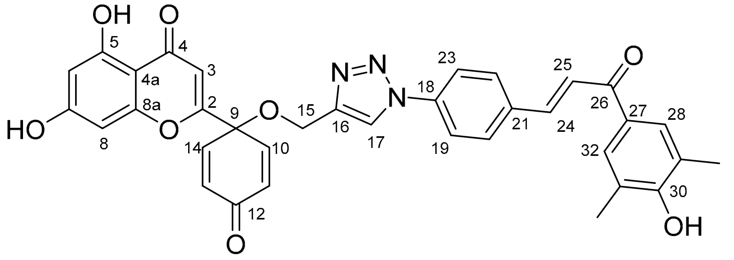

Yellow solid; Yield: 105 mg (55%); ^{1}H-NMR (DMSO-d$_6$): 12.43 (s, 1H, C5OH), 11.28 (br s, 1H, C7OH), 9.29 (br s, 1H, C30OH), 8.99 (s, 1H, H17), 8.11 (d, *J* = 7.9 Hz, 2H, H20 and H22), 7.98 (d, *J* = 15.5 Hz, 1H, H25, overlapped by H19 and H23), 7.97 (d, *J* = 7.9 Hz, 2H, H19 and H23, overlapped by H25), 7.81 (s, 2H, H28 and H32), 7.68 (d, *J* = 15.5 Hz, 1H, H24), 7.16 (d, *J* = 9.7 Hz, 2H, H10 and H14), 6.56 (d, *J* = 9.7 Hz, 2H, H11 and H13), 6.53 (s, 1H, H3), 6.25 (s, 1H, H8), 6.19 (d *J* = 1.5 Hz, 1H, H6), 4.71 (s, 2H, H15), 2.22 (s, 6H, CH_3); ^{13}C-NMR (DMSO-d$_6$): 187.7 (C26), 184.7 (C12), 181.9 (C4), 165.5 (C7), 164.6 (C2), 161.8 (C5), 159.0 (C30), 157.8 (C8a), 145.8 (C10 and C14), 145.2 (C16), 141.4 (C24), 137.8 (C18), 135,7 (C21), 133.2 (C11 and C13), 130.7 (C20 and C22), 130.1 (C28 and C32), 129.3 (C27), 124.7 (C29 and C31), 123.8 (C25), 123.1 (C17), 120.7 (C19 and C23), 108.0 (C3), 104.3 (C4a), 99.8 (C6), 94.5 (C8), 74.8 (C9), 59.0 (C15), 17.1 (CH_3). HRMS: $C_{35}H_{27}N_3O_8$, calculated m/z [M+H$^+$]: 618.18764, found: 618.18341.

Compound **6**

2-(4-(hydroxymethyl)-1*H*-1,2,3-triazol-1-yl)benzaldehyde (**6**)

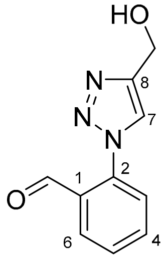

Yellow solid; Yield: 0.52 g (87%); ^{1}H-NMR (CDCl$_3$): 9.87 (s, 1H, CHO), 8.08 (dd, *J* = 7.8 Hz, 0.9 Hz, 1H, H6), 7.94 (s, 1H, H7), 7.74 (td, *J* = 7.6 Hz, 0.9 Hz, H4), 7.64 (t, *J* = 7.6 Hz, 1H, H5), 7.49 (d, *J* = 8.0 Hz, 1H, H3), 4.91 (s, 2H, CH$_2$OH); ^{13}C-NMR (CDCl$_3$): 188.4 (CHO), 148.4 (C8), 138.3 (C2), 134.7 (C4), 130.5 (C1), 130.1 (C5), 129.6 (C6),125.5 (C3), 129.9 (C7), 56.6 (CH$_2$OH).

Compound **8a**

(*E*)-1-ferrocenyl-3-(2-(4-(hydroxymethyl)-1*H*-1,2,3-triazol-1-yl)phenyl)prop-2-en-1-one (**8a**)

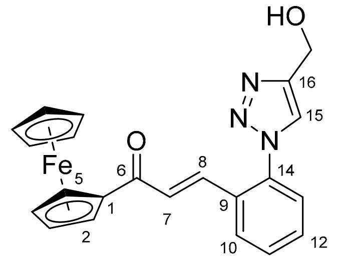

Red solid; Yield: 0.14 g (33%); ^{1}H-NMR (CDCl$_3$): 7.81 (dd, *J* = 7.5 Hz, 0.8 Hz, 1H, H10), 7.79 (s, 1H, H15), 7.56 (td, *J* = 7.6 Hz, 1.0 Hz, H11), 7.51 (td, *J* = 7.5 Hz, 0.9 Hz, 1H, H12), 7.45 (dd, *J* = 8.3 Hz, 1.0 Hz, 1H, H13, overlapped by H8), 7.45 (d, *J* = 8.1 Hz, 1H, H8, overlapped by H13), 6.81 (d, *J* = 15.7 Hz, 1H, H7), 4.88 (br s, 2H, CH$_2$OH), 4.77 (t, *J* = 1.8 Hz, 2H, H2 and H5), 4.55 (t, *J* = 1.8 Hz, 2H, H3 and H4), 4.15 (s, 5H, η^5-C_5H_5); ^{13}C-NMR (CDCl$_3$): 192.6 (C6), 148.3 (C16), 136.3 (C14), 134.6 (C8), 131.3 (C9), 130.5 (C12), 130.2 (C11), 128.4 (C10), 127.0 (C13 and C7), 124.2 (C15), 80.0 (C1), 63.2 (C3 and C4), 70.2 (η^5-C_5H_5), 69.3 (C2 and C5), 56.6 (CH$_2$OH). HRMS: $C_{22}H_{19}FeN_3O_2$, calculated m/z [M+H$^+$]: 414.09049, found: 414.09015.

Compound 8b

(*E*)-1-(4-hydroxy-3,5-dimethylphenyl)-3-(2-(4-(hydroxymethyl)-1*H*-1,2,3-triazol-1-yl)phenyl)prop-2-en-1-one (**8b**)

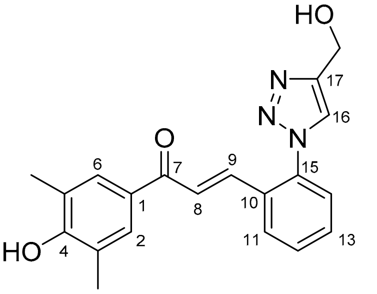

Yellow solid; Yield: 0.02 g (7%); ^{1}H-NMR (DMSO-d$_6$): 9.25 (s, 1H, C4OH), 8.30 (dd, *J* = 7.7 Hz, 1.5 Hz, 1H, H11), 8.36 (s, 1H, H16), 7.85 (d, *J* = 15.5 Hz, 1H, H8), 7.73 (s, 2H, H2 and H6), 7.65 (td, *J* = 7.4 Hz, 1.2 Hz, 1H, H12), 7.61 (td, *J* = 7.3 Hz, 1.6 Hz, 1H, H13), 7.50 (dd, *J* = 7.8 Hz, 1.6 Hz, 1H, H14), 7.45 (d, *J* = 15.5 Hz, 1H, H9), 5.34 (s, 1H, CH$_2$OH), 4.61 (s, 2H, CH$_2$OH), 2.20 (s, 6H, CH$_3$); ^{13}C-NMR (DMSO-d$_6$): 187.5 (C7), 159.0 (C4), 149.0 (C17), 136.9 (C15), 136.4 (C9), 131.1 (C10), 131.4 (C13), 130.6 (C12), 130.2 (C2 and C6), 129.0 (C1), 128.6 (C11), 127.3 (C14), 125.7 (C8 and C16), 124.7 (C3 and C5), 55.4 (CH$_2$OH), 17.0 (CH$_3$). HRMS: $C_{20}H_{19}N_3O_3$, calculated m/z [M+H$^+$]: 350.15047, found: 350.15022.

Compound 8c

(*E*)-3-(4-(4-(hydroxymethyl)-1*H*-1,2,3-triazol-1-yl)phenyl)-1-(3,4,5-trimethoxyphenyl)prop-2-en-1-one (**8c**)

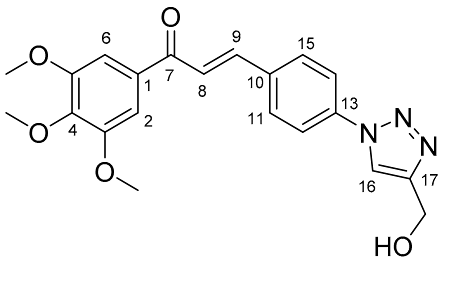

Yellow solid; Yield: 0.23 g (55%); ^{1}H-NMR (DMSO-d$_6$): 8.76 (s, 1H, H16), 8.10 (d, *J* = 8.4 Hz, 2H, H11 and H15), 7.99 (d, *J* = 15.5 Hz, 1H, H8), 7.98 (d, *J* = 8.4 Hz, 2H, H12 and H14), 7.76 (d, *J* = 15.5 Hz, 1H, H9), 7.41 (s, 2H, H2 and H6), 5.34 (t, *J* = 5.5 Hz, 1H, CH$_2$OH), 4.59 (d, *J* = 5.4 Hz, 2H, CH$_2$OH), 3,87 (s, 6H, C3OCH$_3$ and C5OCH$_3$), 3.73 (s, 3H, C4OCH$_3$); ^{13}C-NMR (DMSO-d$_6$): 188.3 (C7), 153.4 (C3 and C5), 149.8 (C17), 142.9 (C9), 142.6 (C4), 138.2 (C13), 135.2 (C10), 133.3 (C1), 131.0 (C11 and C15), 123.2 (C8), 121.4 (C16), 120.4 (C12 and C14), 106.7 (C2 and C6), 60.7 (C4OCH$_3$), 56.7 (C3OCH$_3$ and C5OCH$_3$), 55.4 (CH$_2$OH). HRMS: $C_{21}H_{21}N_3O_5$, calculated m/z [M+H$^+$]: 396.15595, found: 396.15541.

Compound **8d**

(*E*)-1-(4-hydroxy-3,5-dimethylphenyl)-3-(4-(4-(hydroxymethyl)-1*H*-1,2,3-triazol-1-yl)phenyl)prop-2-en-1-one (**8d**)

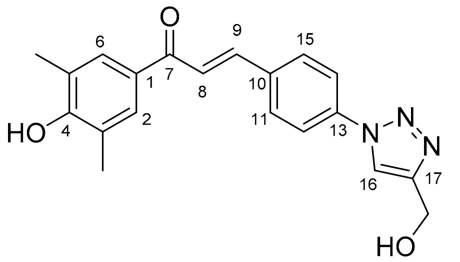

Yellow solid; Yield: 0.03 g (9%); ^{1}H-NMR (DMSO-d$_6$): 9.20 (s, 1H, C4OH), 8.75 (s, 1H, H16), 8.05 (d, *J* = 8.5 Hz, 2H, H11 and H15), 7.97 (d, *J* = 15.6 Hz, 1H, H8, overlapped by H12 and H14), 7.96 (d, *J* = 8.4 Hz, 2H, H12 and H14, overlapped by H8), 7.81 (s, 2H, H2 and H6), 7.68 (d, *J* = 15.6 Hz, 1H, H9), 5.31 (s, 1H, CH_2OH), 4.59 (s, 2H, CH_2OH), 2.22 (s, 6H, CH_3); ^{13}C-NMR (DMSO-d$_6$): 187.7 (C7), 158.9 (C4), 149.8 (C17), 141.4 (C9), 138.0 (C13), 135.5 (C10), 130.7 (C11 and C15), 130.1 (C2 and C6), 129.3 (C1), 124.7 (C3 and C5), 123.6 (C8), 121.4 (C16), 120.4 (C12 and C14), 55.5 (CH2OH), 17.1 (CH_3). HRMS: $C_{20}H_{19}N_3O_3$, calculated m/z [M+H$^+$]: 350.15047, found: 350.15035.

^{1}H-NMR and ^{13}C-NMR spectra of the novel prepared compounds

Figure S1. ^{1}H-NMR spectrum of **3a**

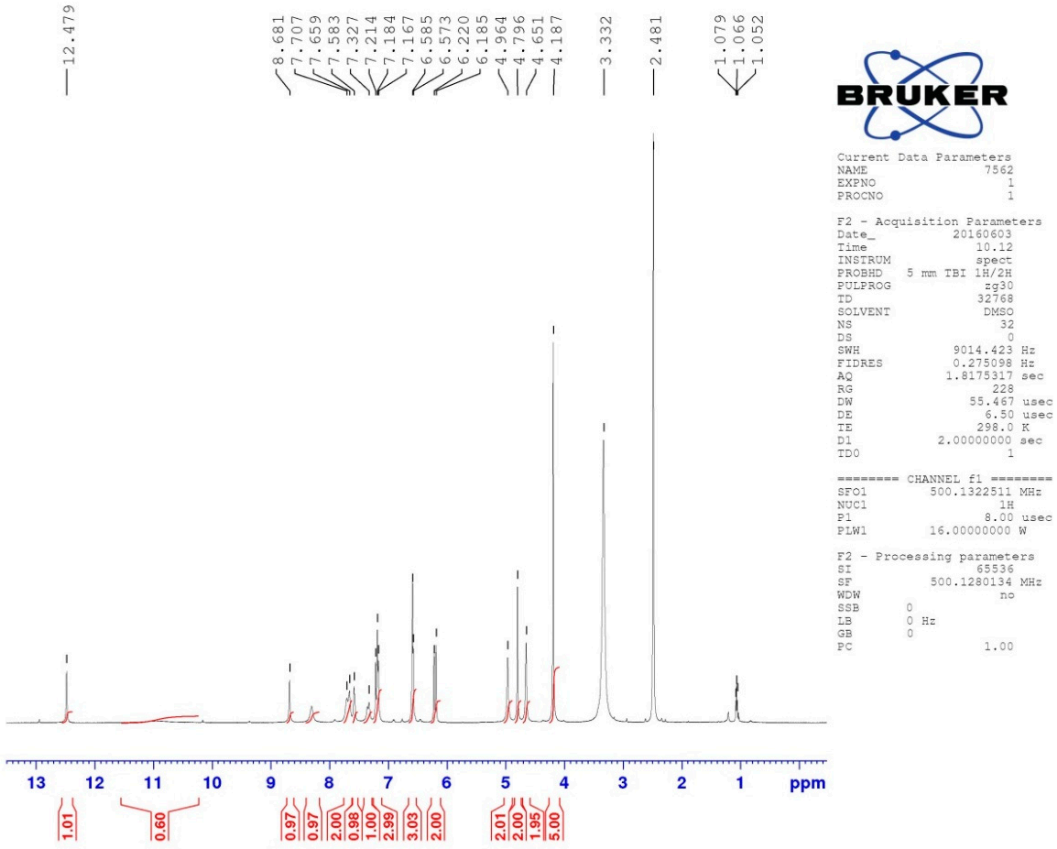

Figure S2. ^{13}C-NMR spectrum of **3a**

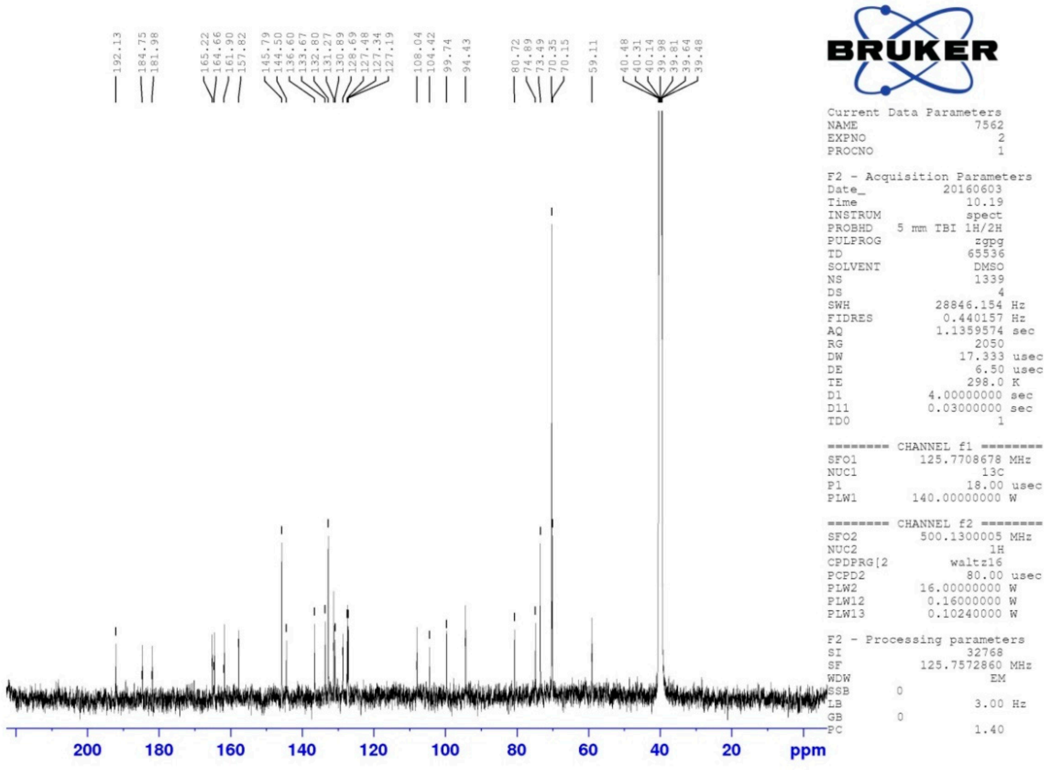

Figure S3. ^{1}H-NMR spectra of **3b**

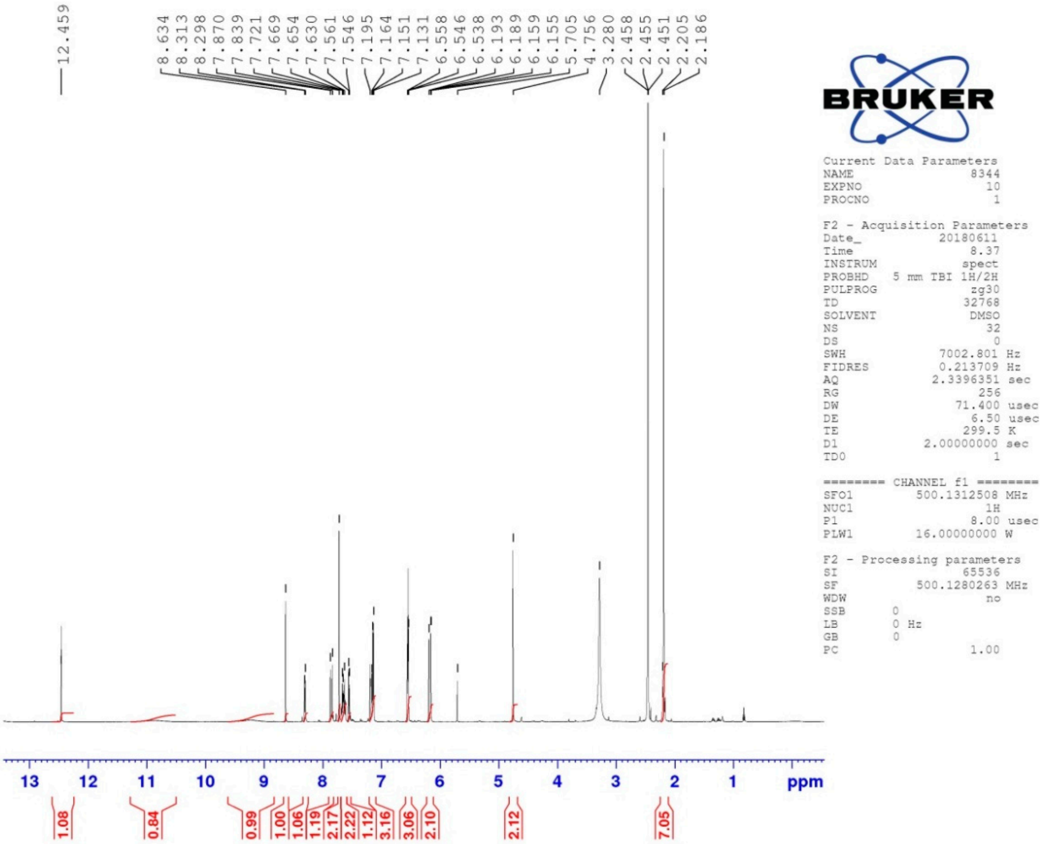

Figure S4. ^{13}C-NMR spectra of **3b**

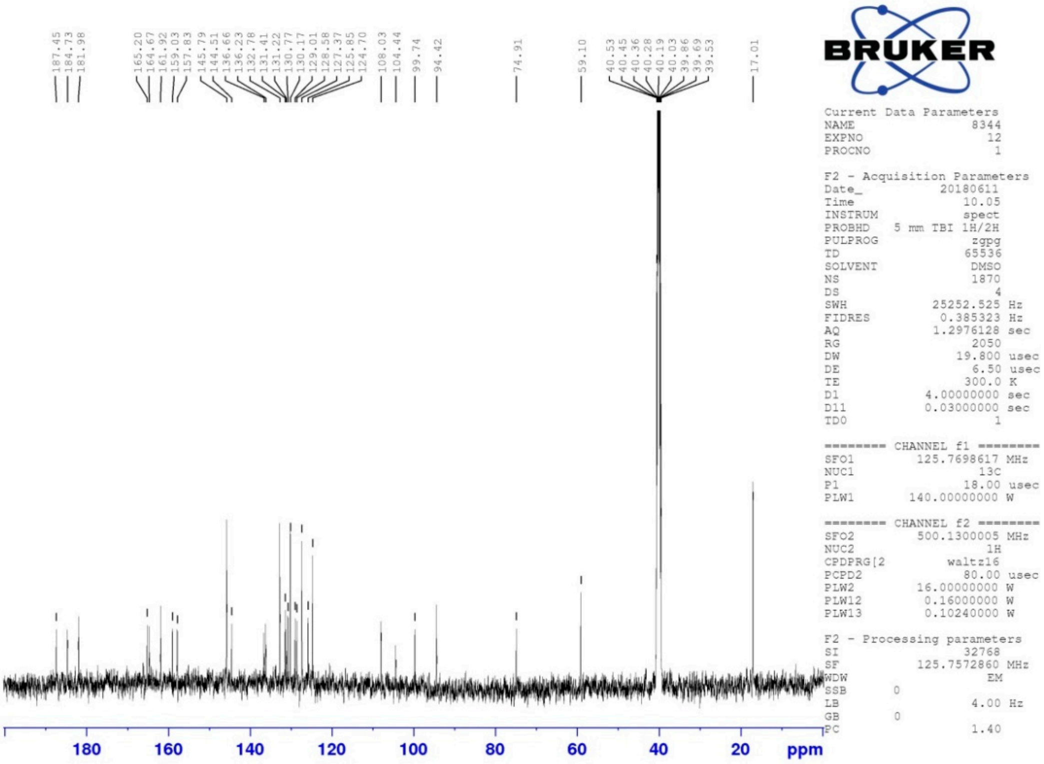

Figure S5. ^{1}H-NMR spectra of **3c**

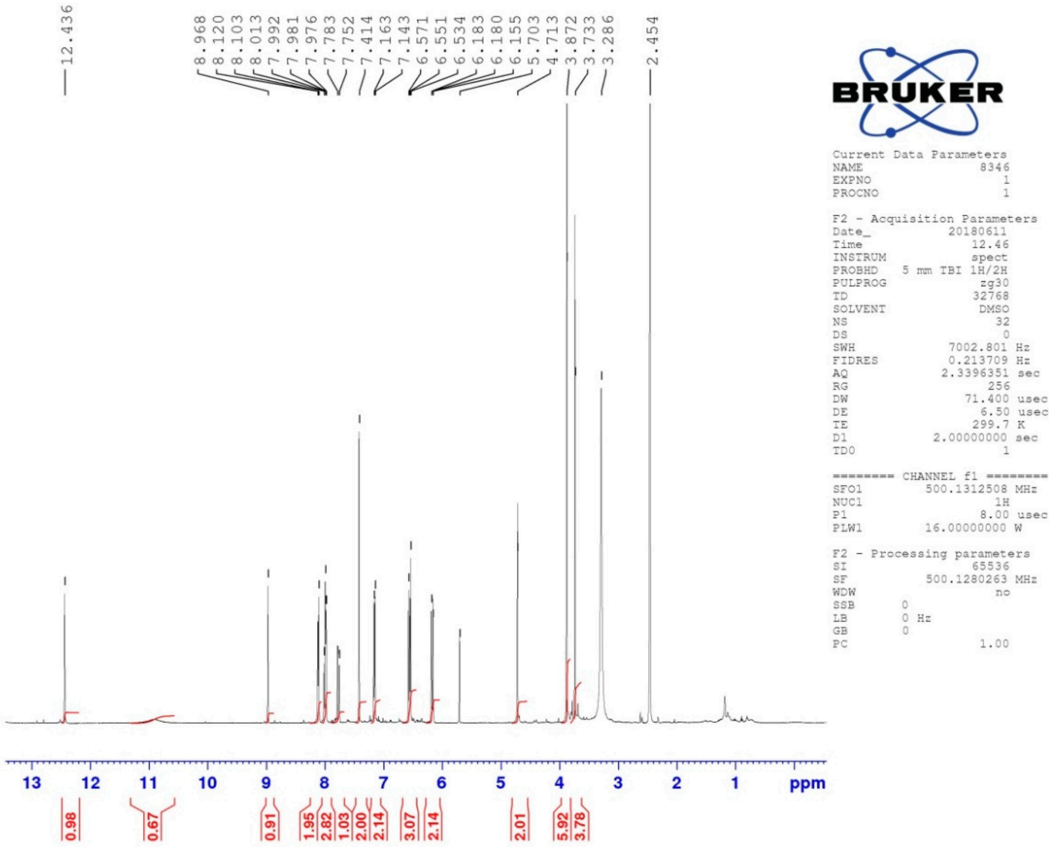

Figure S6. ^{13}C-NMR spectra of **3c**

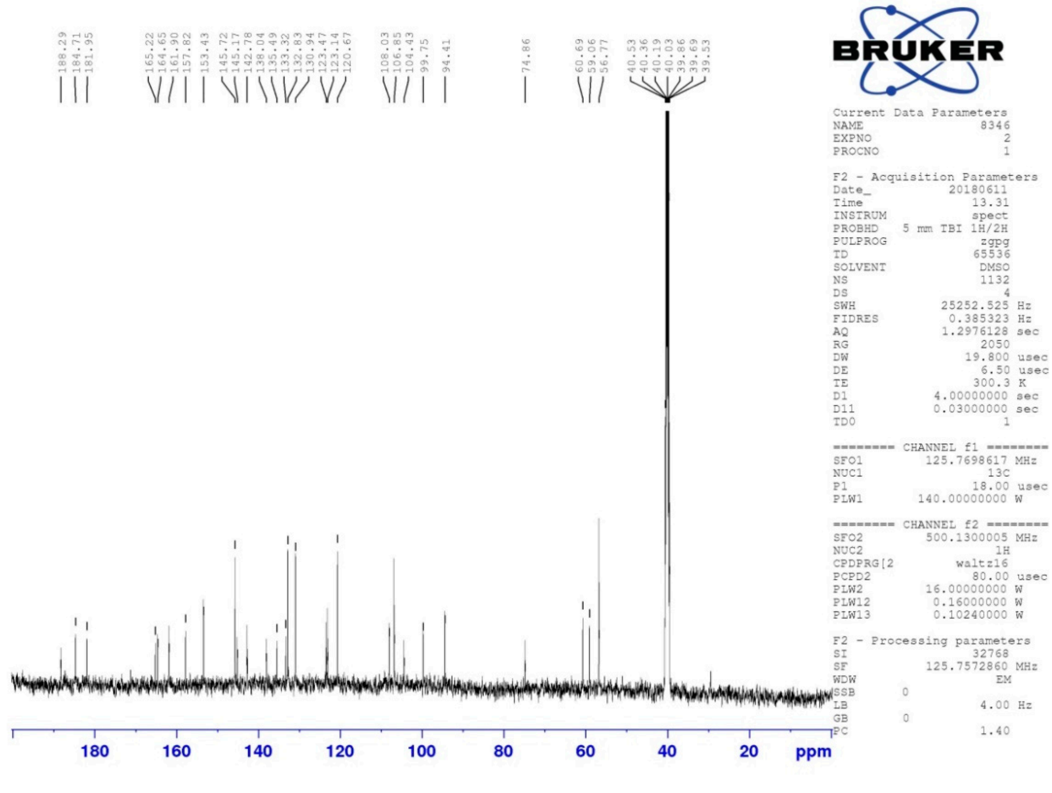

Figure S7. ^{1}H-NMR spectra of **3d**

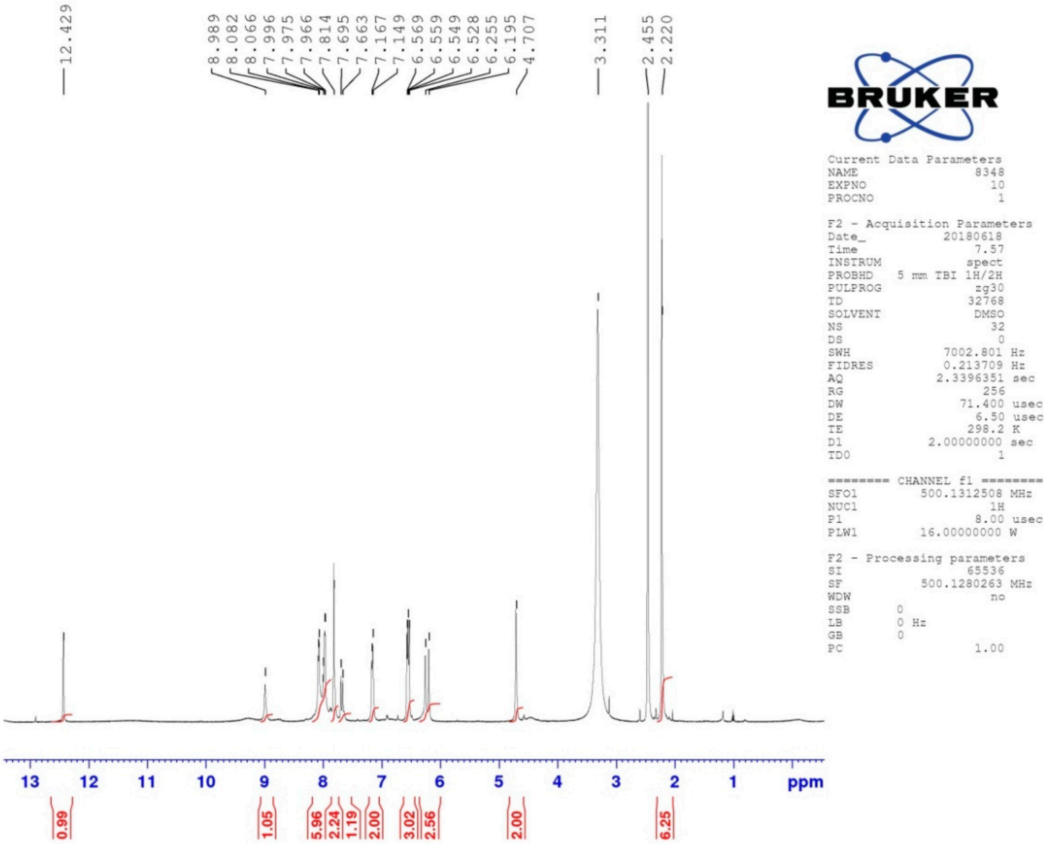

Figure S8. ^{13}C-NMR spectra of **3d**

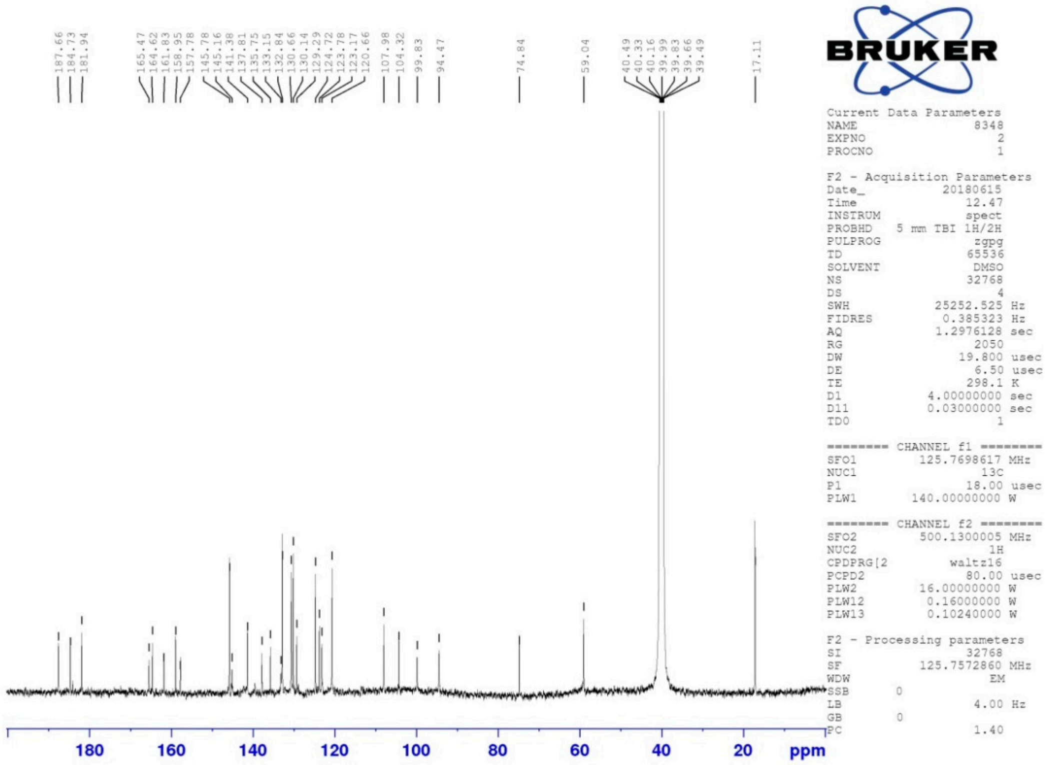

Figure S9. ^{1}H-NMR spectra of **6**

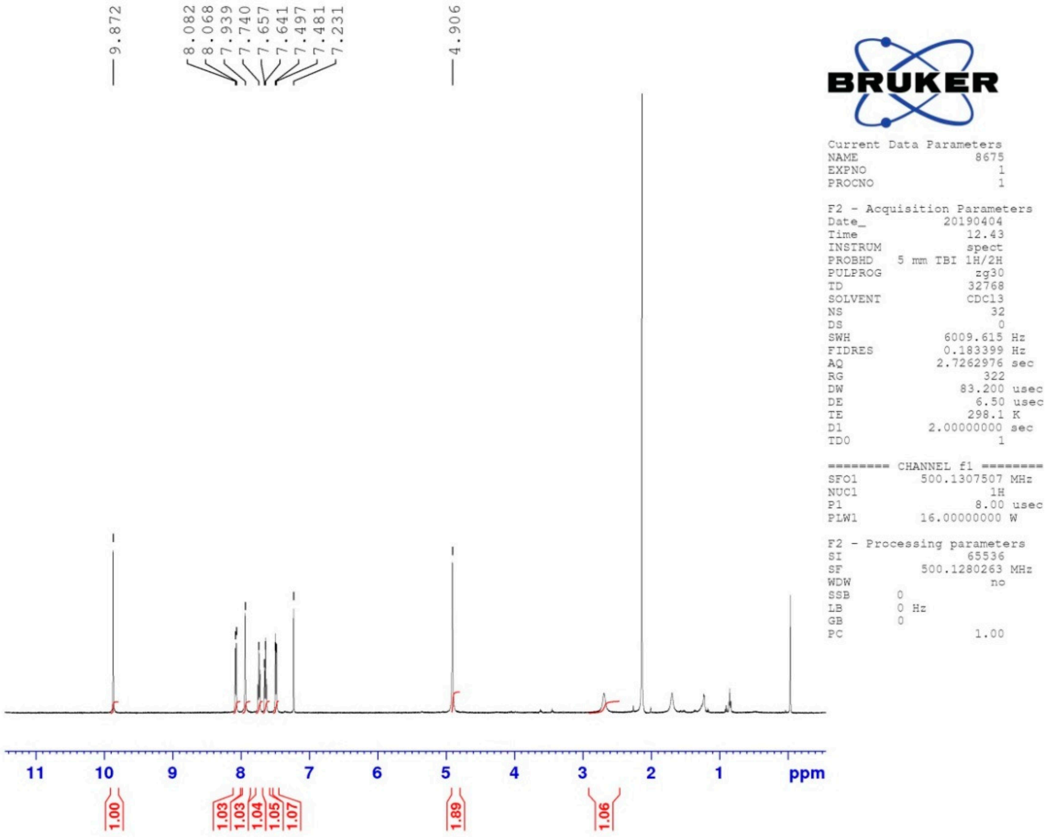

Figure S10. ^{13}C-NMR spectra of **6**

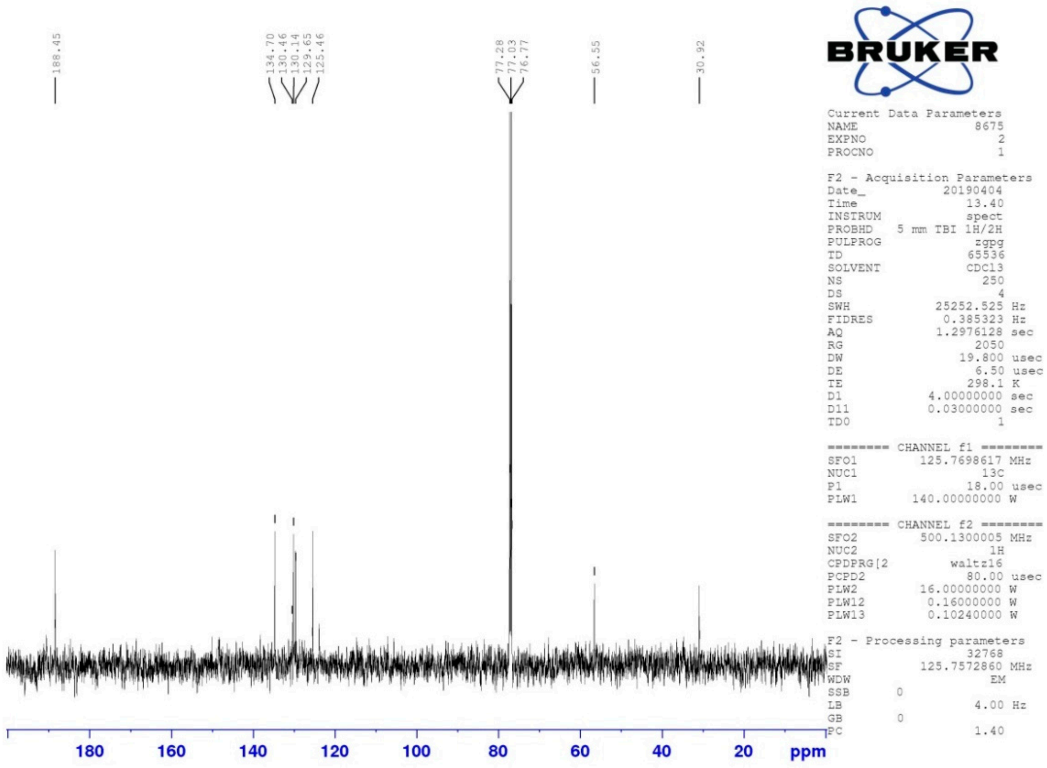

Figure S11. ^{1}H-NMR spectra of **8a**

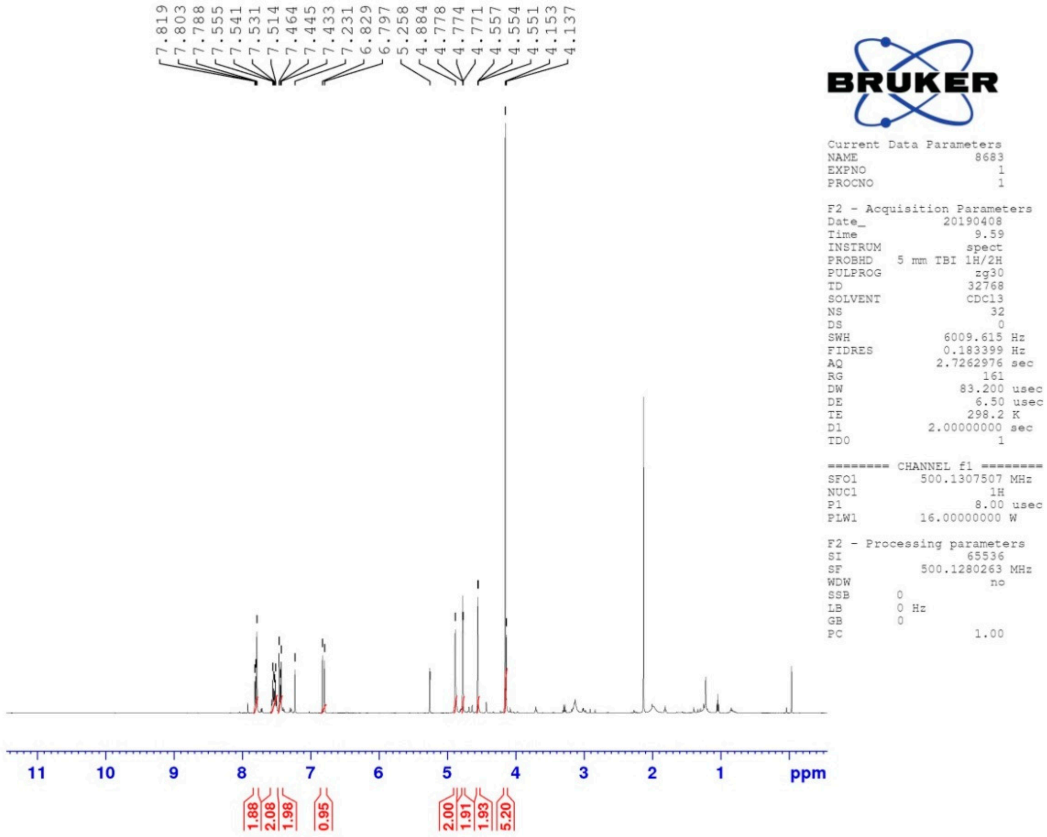

Figure S12. ^{13}C-NMR spectra of **8a**

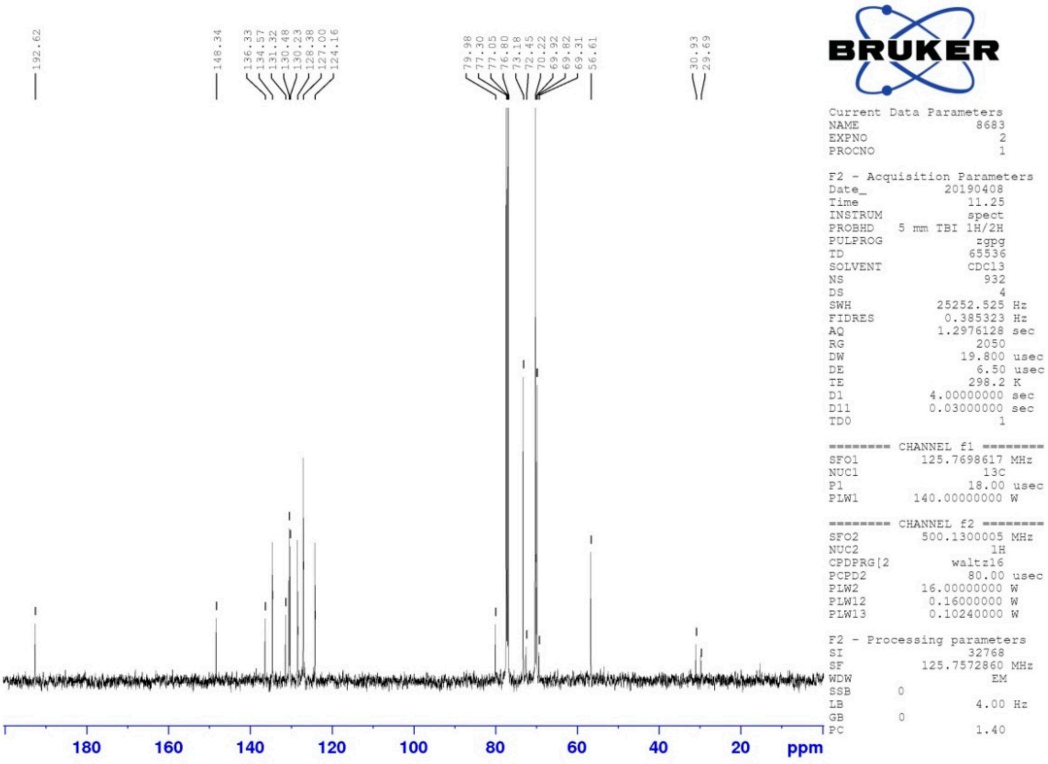

Figure S13. ^{1}H-NMR spectra of **8b**

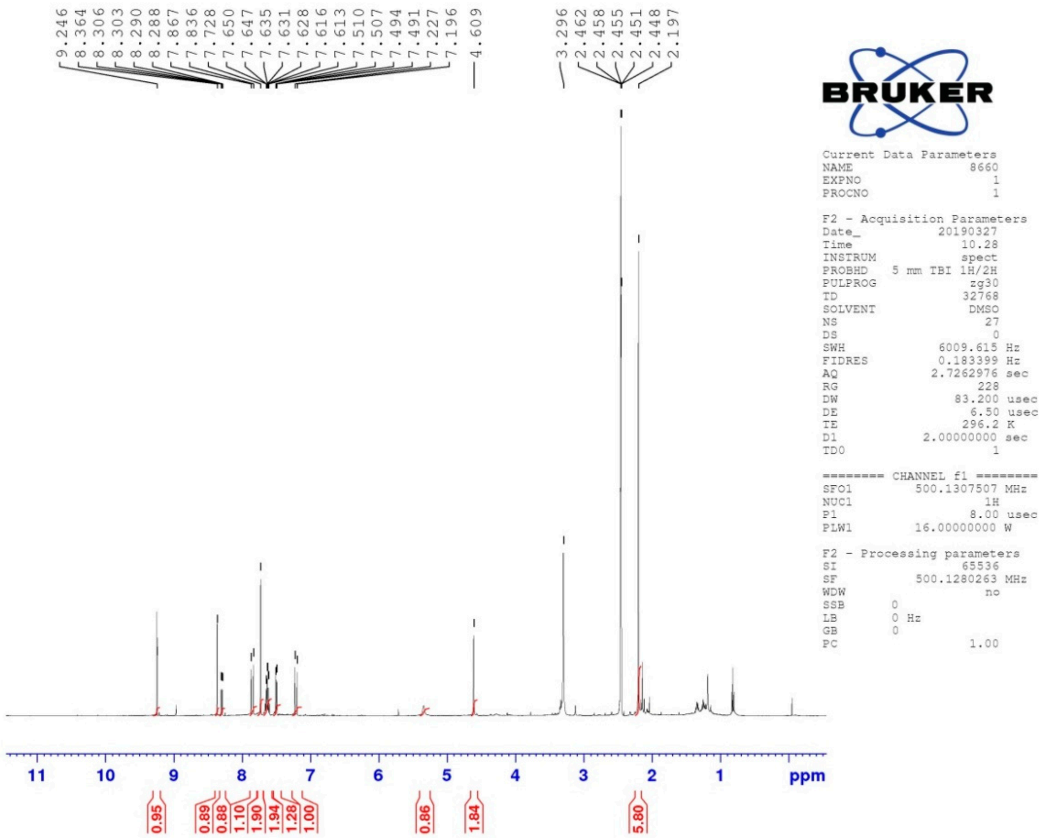

Figure S14. ^{13}C-NMR spectra of **8b**

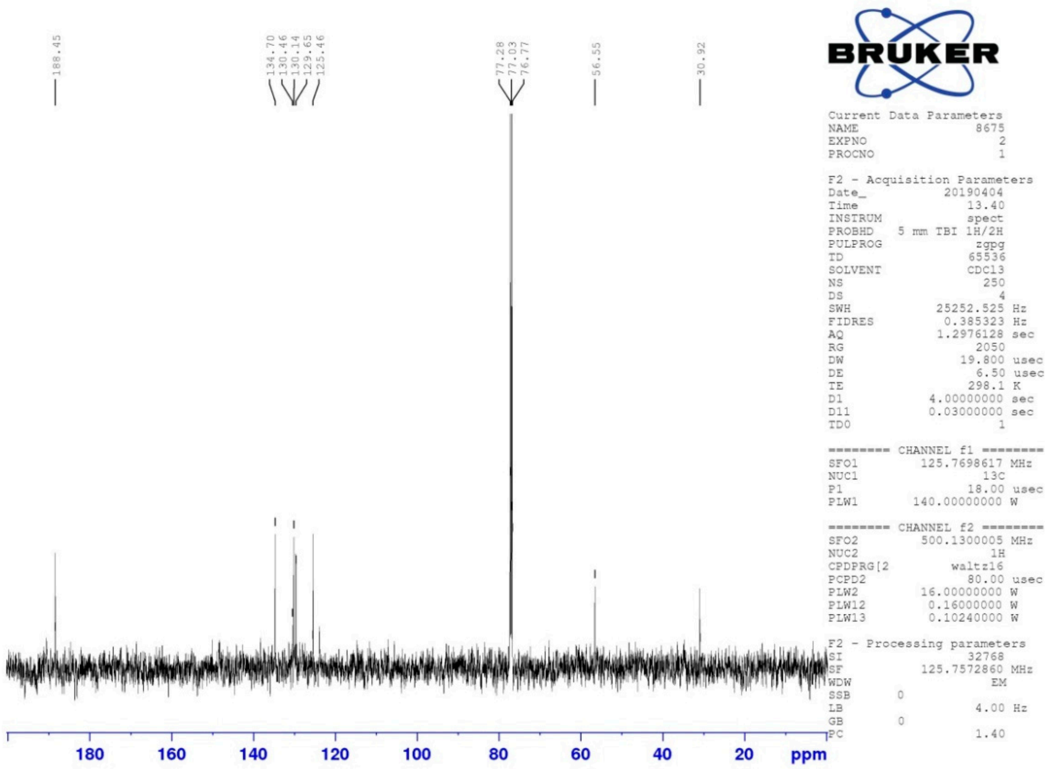

Figure S15. ^{1}H-NMR spectra of **8c**

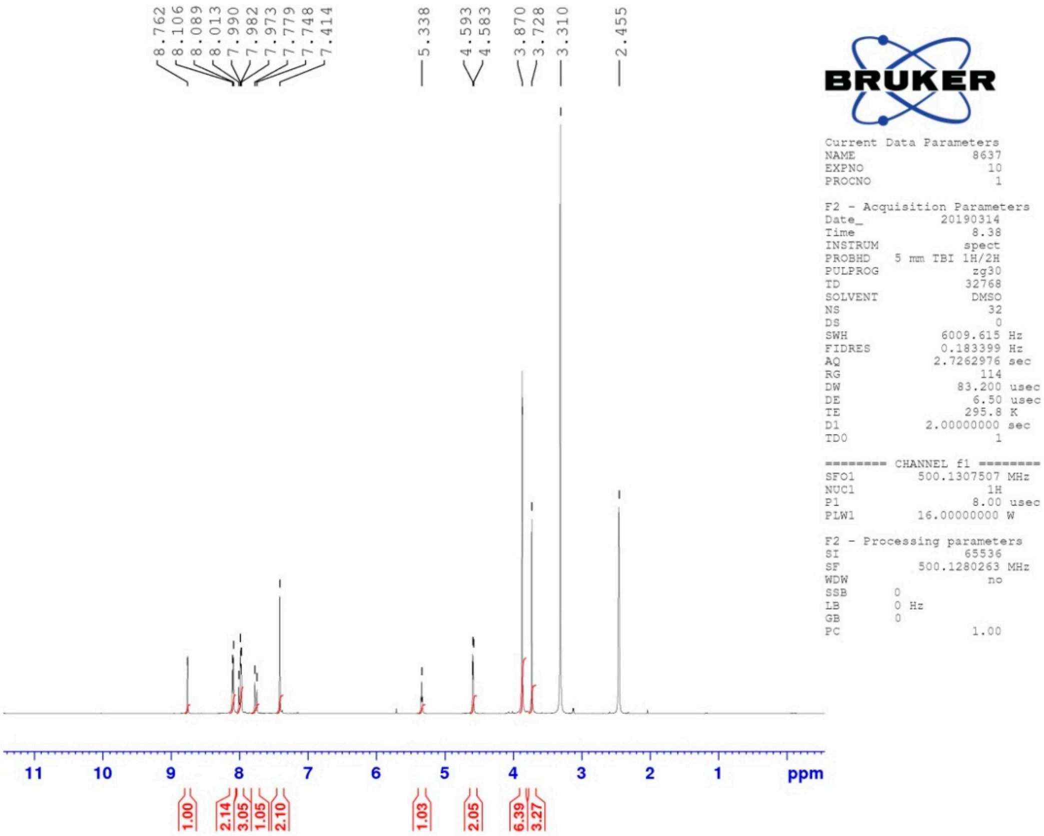

Figure S16. ^{13}C-NMR spectra of **8c**

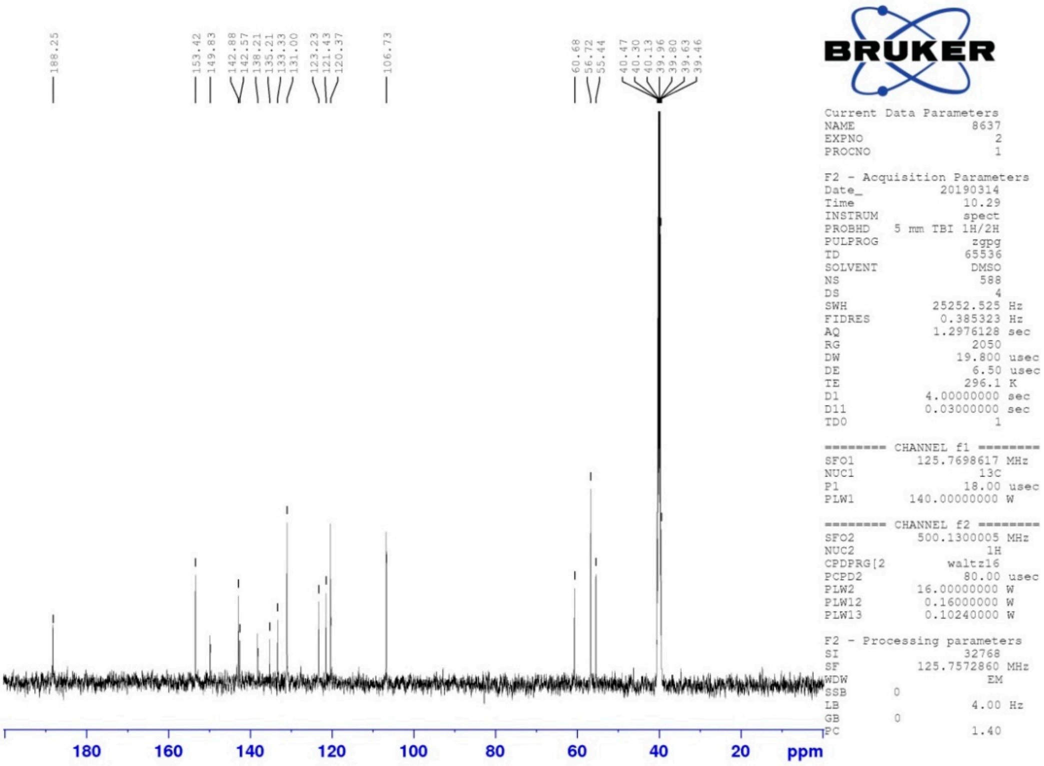

Figure S17. ^{1}H-NMR spectra of **8d**

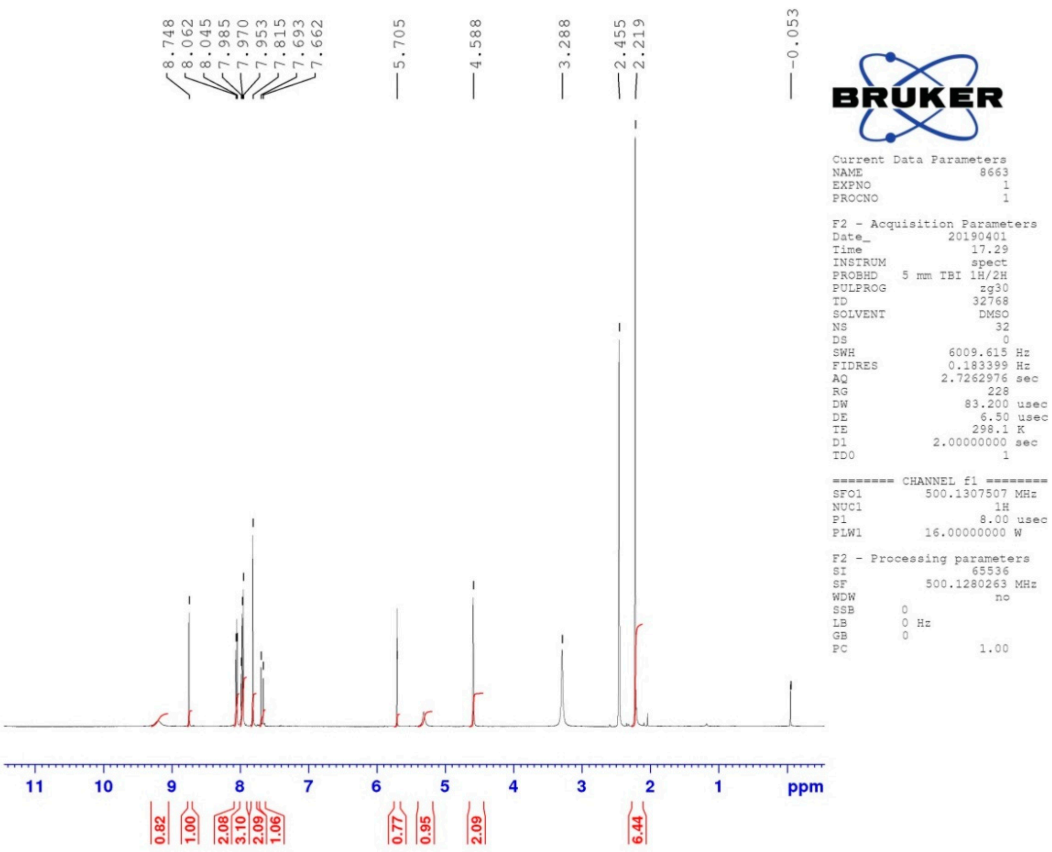

Figure S18. ^{13}C-NMR spectra of **8d**

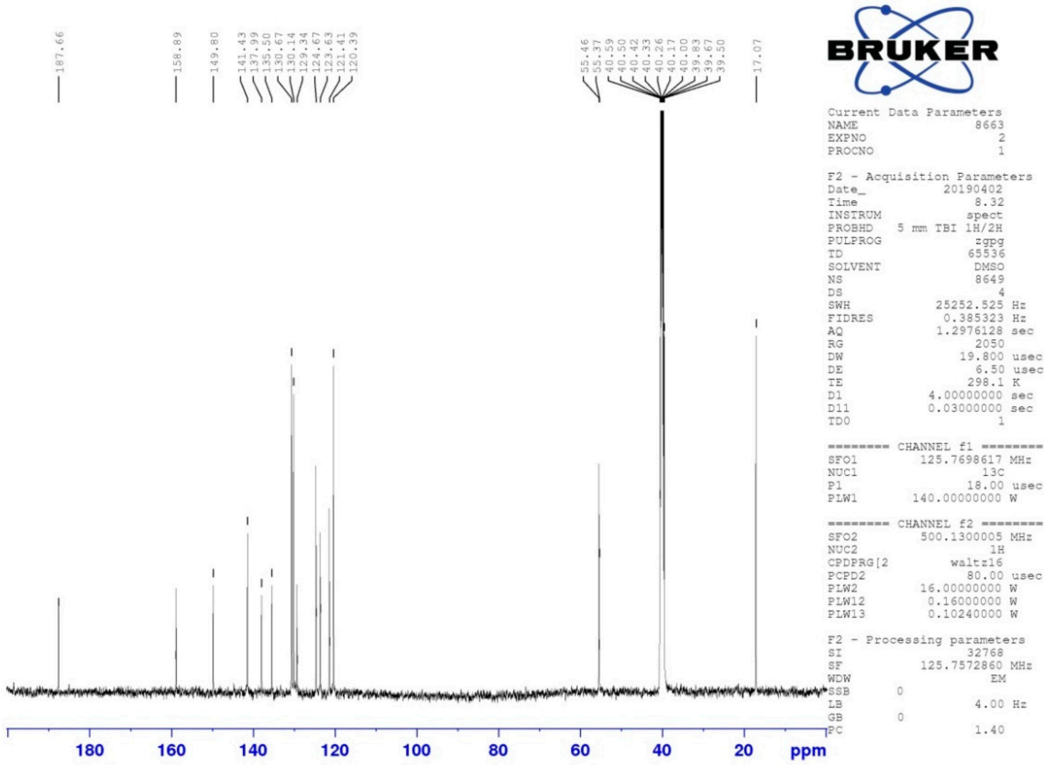

Figure S19. Background corrected fluorescent emission spectra of compounds 3a-d obtained upon exciting molecules at 488 nm.

Symbols **a-d** represent compounds **3a-d**, respectively. Based on the spectra, no spectral interference with the bioassays' results can be expected.

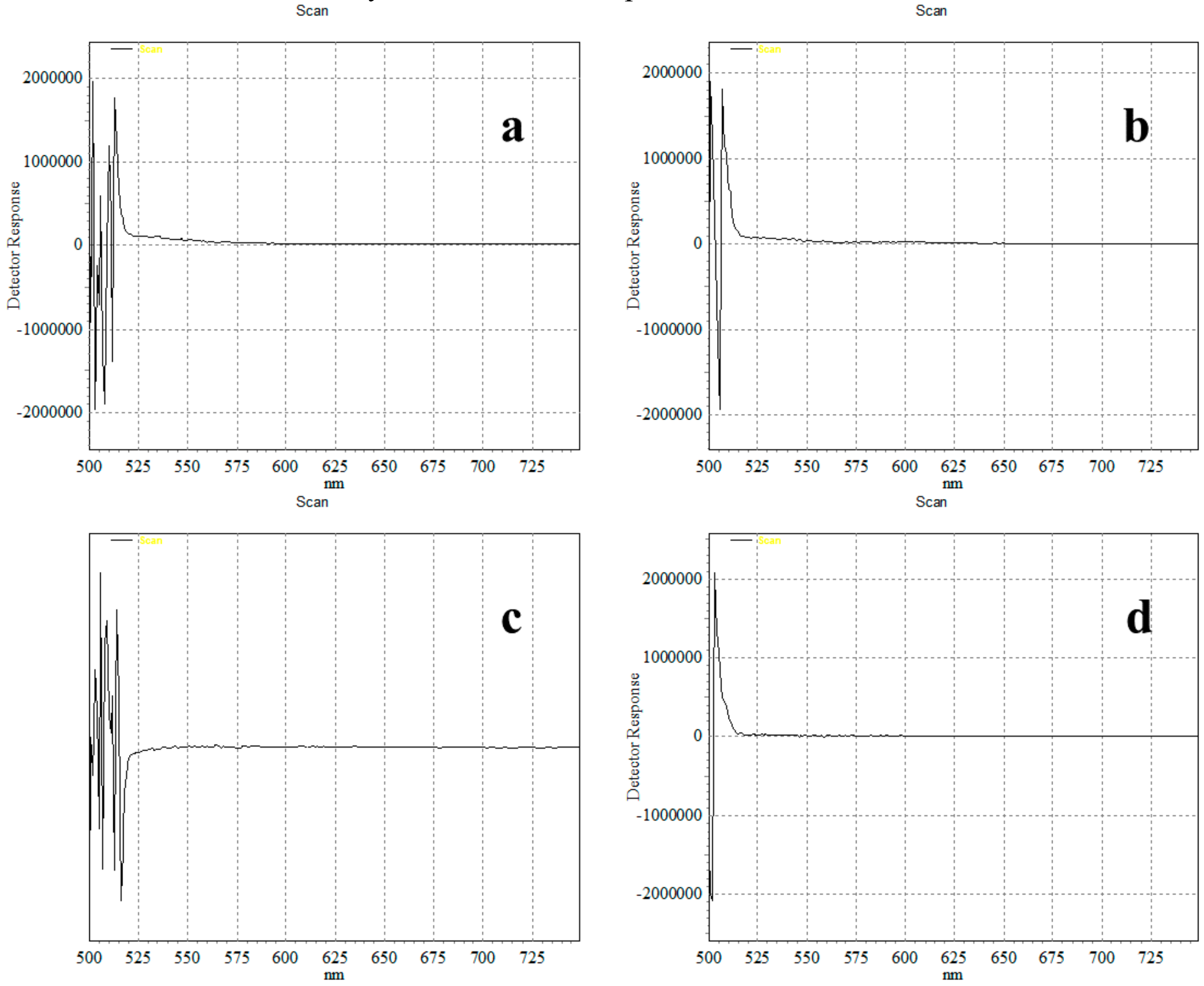

Figure S20. Effect of compound 3c on the cell cycle distribution of MDA-MB-231 cells. Cells were treated for 24h; *, ** and ***: $p < 0.05$, $p < 0.01$ and $p < 0.001$, respectively, by means of one-way ANOVA followed by Dunnett's post-hoc test.

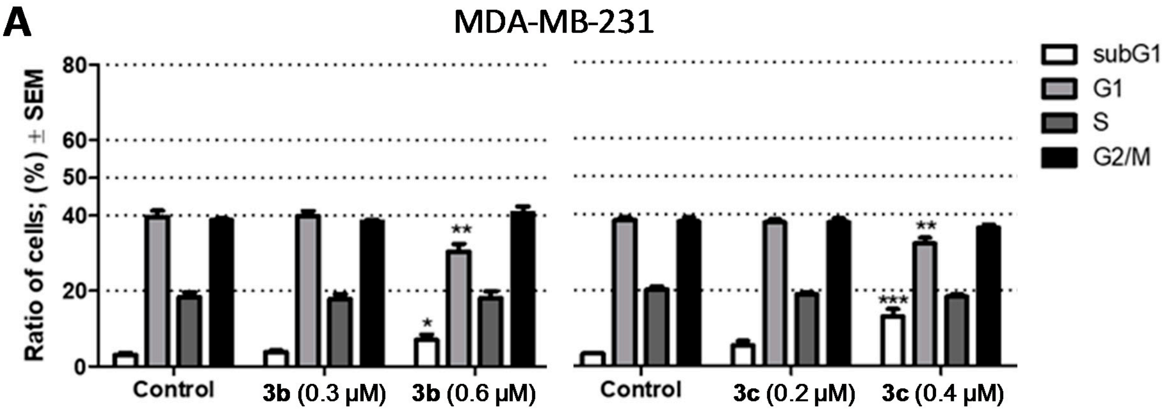

Figure S21. Effect of compound 3c on the caspase-3 activity of MDA-MB-231 cells. Cells were treated for 24h or 48h. *, and ***: $p < 0.05$, and $p < 0.001$, respectively, by means of one-way ANOVA followed by Dunnett's post-hoc test. Cells were treated for 24 h (**A**) or 48 h (**B**), and a dose and time dependent increase of caspase-3 activity was observed.

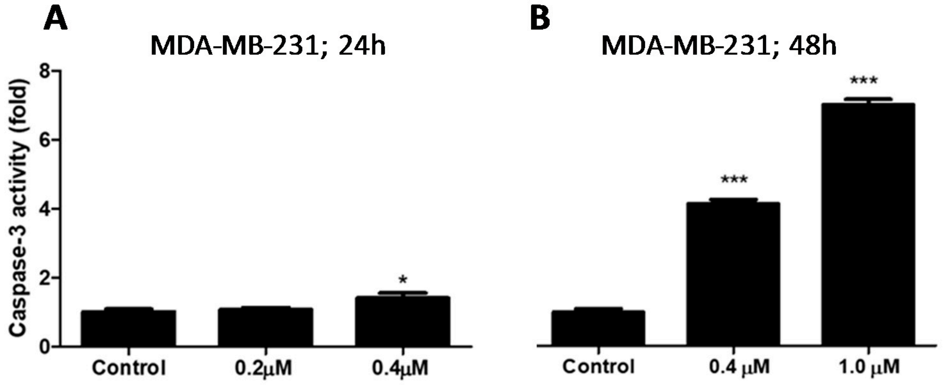

Supplement: Supplementary file 1 [file antioxidants-09-00519-s001.pdf]
